# Coupling of *in vitro* Neocortical-Hippocampal Coculture Bursts Induces Different Spike Rhythms in Individual Networks

**DOI:** 10.3389/fnins.2022.873664

**Published:** 2022-05-23

**Authors:** ChihHsiang Chang, Takuma Furukawa, Takahiro Asahina, Kenta Shimba, Kiyoshi Kotani, Yasuhiko Jimbo

**Affiliations:** ^1^Department of Precision Engineering, School of Engineering, The University of Tokyo, Tokyo, Japan; ^2^Research Center for Advanced Science and Technology, The University of Tokyo, Tokyo, Japan

**Keywords:** hippocampus, neocortex, micro-tunnel, coculture (co-culture), micro-electrode array (MEA), burst activity coupling

## Abstract

Brain-state alternation is important for long-term memory formation. Each brain state can be identified with a specific process in memory formation, e.g., encoding during wakefulness or consolidation during sleeping. The hippocampal-neocortical dialogue was proposed as a hypothetical framework for systems consolidation, which features different cross-frequency couplings between the hippocampus and distributed neocortical regions in different brain states. Despite evidence supporting this hypothesis, little has been reported about how information is processed with shifts in brain states. To address this gap, we developed an *in vitro* neocortical-hippocampal coculture model to study how activity coupling can affect connections between coupled networks. Neocortical and hippocampal neurons were cultured in two different compartments connected by a micro-tunnel structure. The network activity of the coculture model was recorded by microelectrode arrays underlying the substrate. Rhythmic bursting was observed in the spontaneous activity and electrical evoked responses. Rhythmic bursting activity in one compartment could couple to that in the other via axons passing through the micro-tunnels. Two types of coupling patterns were observed: slow-burst coupling (neocortex at 0.1–0.5 Hz and hippocampus at 1 Hz) and fast burst coupling (neocortex at 20–40 Hz and hippocampus at 4–10 Hz). The network activity showed greater synchronicity in the slow-burst coupling, as indicated by changes in the burstiness index. Network synchronicity analysis suggests the presence of different information processing states under different burst activity coupling patterns. Our results suggest that the hippocampal-neocortical coculture model possesses multiple modes of burst activity coupling between the cortical and hippocampal parts. With the addition of external stimulation, the neocortical-hippocampal network model we developed can elucidate the influence of state shifts on information processing.

## Introduction

Long-term memory formation is a multi-step process that includes steps of encoding and consolidation/reconsolidation, which occur at different times and in different brain states. In memory encoding, new information learned during wakefulness is encoded and temporarily stored in the hippocampus. Subsequently, during sleep, this temporary and labile trace is transformed into a stable and long-lasting form in the memory consolidation step. Through this process, called systems consolidation, memories initially dependent on the hippocampus are progressively reorganized through time; the hippocampus gradually becomes less important for storage and retrieval, and a more permanent memory develops in distributed regions of the neocortex (Squire et al., 2015; Klinzing et al., 2019).

To explain the process of systems consolidation and the transfer of information from the hippocampus to the neocortex, György Buzsáki proposed the hippocampal (HPC)-neocortical (CX) dialogue model (Buzsáki, 1996). The model describes the hippocampus as a fast-learning system that acquires new information during wakefulness, and the neocortex as a “slow learner” that learns information processed by the hippocampus during slow-wave sleep. According to the HPC-CX dialogue model, the low-frequency wave in the hippocampus (receiver) couples with the high-frequency wave in neocortical regions (sender) during wakefulness. During sleep, these roles are reversed, as the high-frequency wave in the hippocampus (sender) couples with the low-frequency wave in the neocortical regions (receiver). This hypothesis is supported by neurocomputational models (McClelland, 2013), electroencephalography, and functional magnetic resonance imaging in humans (Mitra et al., 2016; Ngo et al., 2020). Although we find extensive studies on the effect of frequency-specific network coupling on different states/stages of memory formation and on behaviors, little has been reported about how the observed couplings between waveforms alter neuronal connections at the subnetwork and cellular levels.

To study how activity coupling can affect information transfer between networks, we used the *in vitro* co-culture method. Due to their ability to preserve the cellular characteristics of the original structures, *in vitro* co-culture models have been used to study pathophysiology at the sub-circuit level without interference from other neuronal populations (Kanagasabapathi et al., 2012; Sarkar et al., 2018; Virlogeux et al., 2018; Pelkonen et al., 2020). Additionally, cocultured HPC subregions have shown results supporting hypotheses on information processing such as information encoding/decoding (Poli et al., 2017) and pattern separation/completion (Poli et al., 2018) through the HPC trisynaptic pathway.

In the present study, an *in vitro* CX-HPC coculture model was used to evaluate how different modes of inter-regional coupling can affect neuronal connections during systems consolidation. The results on spontaneous activities and evoked responses showed two types of activity coupling between CX and HPC networks across samples: (1) slow-burst coupling, wherein CX networks expressed a burst firing rhythm of 0.1–0.5 Hz and HPC networks expressed a burst firing rhythm of 1 Hz; and (2) fast burst coupling, wherein CX networks expressed firing activity of 20–40 Hz and HPC networks expressed a burst firing rhythm of 4–10 Hz. Additionally, CX subnetworks showed different levels of synchronicity in each coupling type. Finally, the evoked cross-chamber responses showed a higher response rate in the CX-to-HPC direction.

## Materials and Methods

### Design of the Hippocampal-Neocortical Coculture Device

To study the interaction between CX and HPC networks, the developed model should preserve the intrinsic cellular properties of both networks and allow communication between heterogeneous cultures. Photolithographic techniques were used to construct chamber structures for network separation and particularly micro-tunnel structures for axonal passage, to allow inter-network communication. A micro-electrode array (MEA) substrate, also fabricated photolithographically, was incorporated into the design to permit the recording of neural activities.

**Figure 1:**
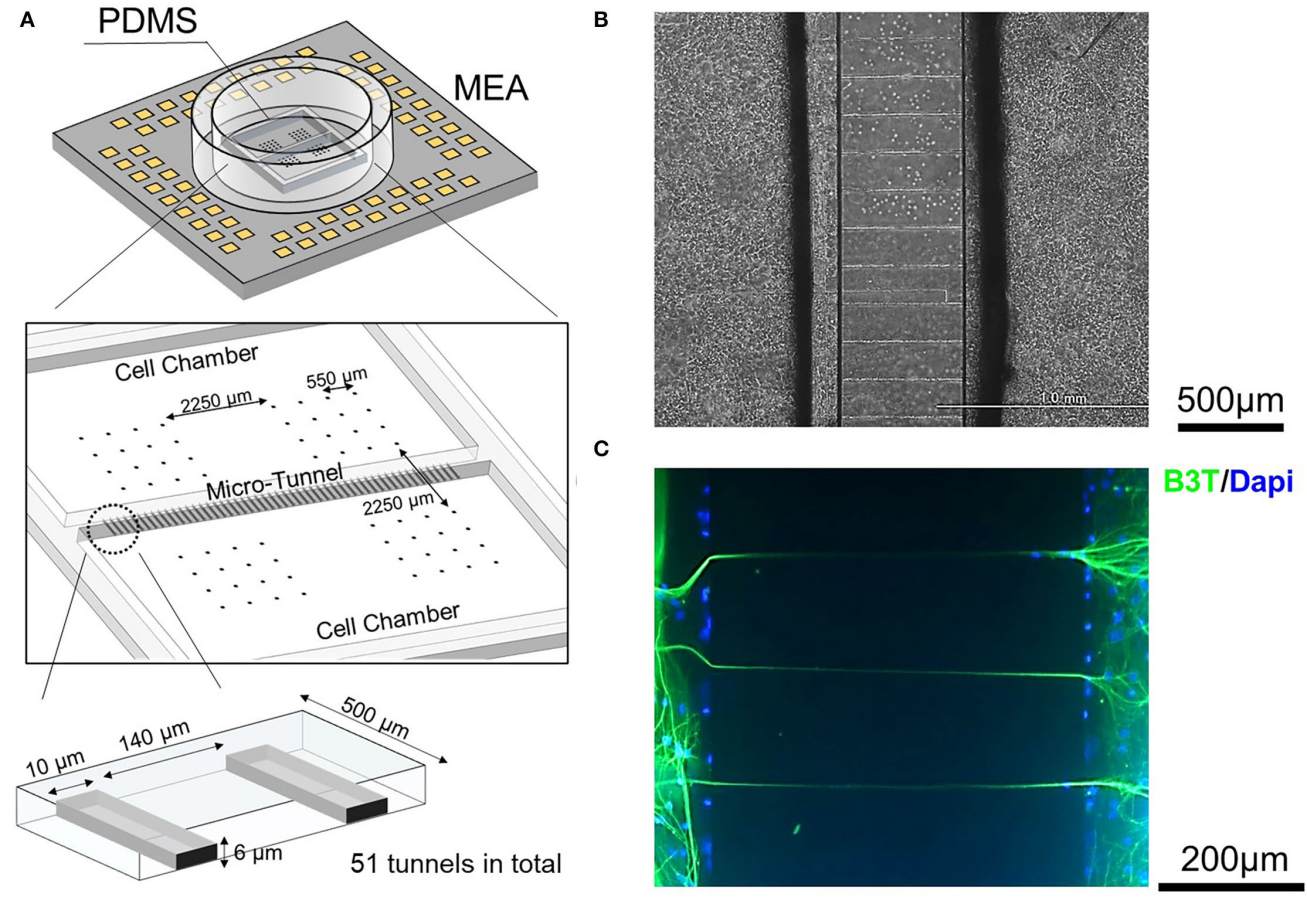
Design of the hippocampal (HPC)-neocortical (CX) coculture device and confirmation of micro-tunnel function. **(A)** A schematic of the chamber/micro-tunnel structure fabricated from polydimethylsiloxane (PDMS) combined with the micro-electrode array (MEA) substrate. The two cell-culture compartments are connected by 51 micro-tunnels to allow inter-network communication. **(B)** Micro-tunnels were observed by phase-contrast microscopy. **(C)** Micro-tunnel function confirmed by immunocytochemistry. The presence of axons in the micro-tunnels after culturing is confirmed by Beta III Tubulin (B3T) immunopositivity, shown in green. Nuclei are stained with DAPI and appear in blue in the figure. The dimensions of the micro-tunnels were chosen to allow the passage of axons while preventing the entrance of cell bodies.

Figure 1A shows a schematic diagram of a polydimethylsiloxane (PDMS) structure consisting of two soma chambers of 9 mm × 5 mm. The chambers are connected by 51 micro-tunnels having a height of 6 μm, a width of 10 μm, and a length of 500 μm, to allow inter-network activity propagation. The dimensions of the micro-tunnels were chosen to allow axon passage but limit the entry of cell bodies, to prevent the mixing of neurons derived from different brain regions (Pan et al., 2015; DeMarse et al., 2016).

Chamber and micro-tunnel structures were made using PDMS (SYLGARDTM 184 Silicone Elastomer; DOW, Midland, MI, United States) (SYLGARD™ 184 Silicone Elastomer; DOW) as in previous studies (Taylor et al., 2005; Shimba et al., 2019). The primary mold was made using two-layer photolithography. The first layer had a height of 6 μm, to implement the micro-tunnel structure, and was made of SU-8 3005 photoresist (MicroChem, Westborough, MA, United States), and the second layer had a height of 100 μm and was made of SU-8 3050 photoresist (MicroChem, Westborough, MA, United States), all using standard photolithographic techniques. Next, PDMS and its curing reagent were mixed in a ratio of 10:1 (10 bases and 1 curing agent), poured onto the mold, and cured by oven-baking at 80°C for 60 min. Finally, the PDMS casting was detached from the mold and shaped using scalpels.

The MEA was fabricated using a previously described method (Shimba et al., 2019). First, an electrical circuit was fabricated by chemically etching an indium-tin-oxide (ITO)-coated glass substrate with an etchant, ITO-02 (Kanto Chemical Co. Inc., Tokyo, Japan). Then the positive photoresist OFPR (800 LB, 23 cp; Tokyo Ohka Kogyo Co. Ltd., Tokyo, Japan) was coated onto the ITO glass substrate as an insulation layer and developed using NMD-3 (Tokyo Ohka Kogyo Co. Ltd., Tokyo, Japan) to expose the electrodes and external connection pads. A glass ring with an inner and outer diameter of 18 and 22 mm, respectively, was attached to the substrate with silicone (KE-103; Shin-Etsu Chemical Co., Ltd., Tokyo, Japan). Finally, platinum black was deposited on the electrodes. The electrodes were arranged into four areas as shown in Figure 1A to record overall network activity. Each area contained 16 electrodes (50 × 50 μm in size, 550 μm spacing, square grid pattern) with an inter-area space of 2,250 μm.

Before assembling the device, the PDMS structure and micro-tunnels were coated with 3% bovine serum albumin (Sigma, St. Louis, MO, United States) at 4°C overnight. Meanwhile, the MEA substrates were coated with 0.1% polyethyleneimine (Sigma, St. Louis, MO, United States) in borate buffer at 4°C overnight and rinsed with sterilized water four times, then coated in laminin. Alignment of the PDMS on the MEA substrates was performed under a microscope. The parts were then pressed using tweezers to ensure good contact between them. A total of 32 electrodes underlay each soma chamber. After assembly, Dulbecco's phosphate-buffered saline (D-PBS, FUJIFILM Wako Pure Chemical, Osaka, Japan) was added to the soma chambers, which were then rinsed with sterilized water once before laminin coating.

### Preparation of the Hippocampal/Neocortical Primary Culture

Before cell plating, soma chambers and micro-tunnels were coated with 20 μg/mL laminin (Thermo Fisher, Waltham, MA, United States) in D-PBS for 1 h in an incubator. HPC and CX neurons were dissected from Wistar rat 19-day embryos (Oriental Yeast, Tokyo, Japan). The dissected brain structures were treated with 0.5% trypsin at 37°C for 17 min for CX tissues and for 15 min for HPC tissues. The brain tissues were then rinsed with DMEM supplemented with 10% FBS, 100 units/mL penicillin, and 100 μg/mL streptomycin (All from Thermo Fisher, Waltham, MA, United States), to stop enzyme action. Following enzyme treatment, the brain tissues were further physically dissociated by pipetting. The dissociated cells were suspended in a Neurobasal medium supplemented with B-27 supplement, 2 mM GlutaMAX, 100 units/mL penicillin, and 100 μg/mL streptomycin (All from Thermo Fisher, Waltham, MA, United States). Furthermore 50 μL of cell suspension were plated into each soma chamber, with a final concentration of 5,000 cells per mm^2^ in each chamber. A mixture of Neurobasal medium and conditioned medium (1:1) was added for the first week. The conditioned medium was based on a Neurobasal medium and derived from mature CX monocultures after 28 days *in vitro*. For the following weeks, half of the Neurobasal medium with supplement was changed twice a week. The cells were cultured under constant conditions (37°C, 5% CO_2_) in a humidified cell-culture incubator (BNA600, Yamato Scientific Co., Ltd., Tokyo, Japan). Supplementary Figure 1 shows phase-contrast micrographs of CX and HPC cultures taken from different samples on different days *in vitro*.

There were two types of coculture models which were used in this study, as defined by whether the networks cultured on the two sides of the device were derived from the same or different parts of the brain. Models with two networks derived from the same brain region were defined as homogeneous, e.g., CX-CX coculture or HPC-HPC coculture. Models with networks derived from different brain regions were defined as heterogeneous, e.g., CX-HPC coculture. Six CX homogeneous models and six HPC homogeneous models were recorded as controls, while 23 heterogeneous models were recorded as the condition of interest. Figure 1B shows a phase contrast micrograph of cultured neurons in the vicinity of a micro-tunnel.

All procedures were approved by the local ethics committee of the University of Tokyo.

### Immunocytochemistry

Immunocytochemistry was performed to visualize the morphological structures of cultured cells. First, the culture was fixed in 4% paraformaldehyde/phosphate buffer (FUJIFILM Wako Pure Chemical, Osaka, Japan) at room temperature for 30 min. The culture was then permeabilized and blocked using 4% Block Ace (KAC Co. Ltd, Tokyo, Japan) and 0.25% Triton X-100 in PBS (Thermo Fisher, Waltham, MA, United States) at 4°C overnight. The culture was then incubated with primary antibodies in 0.4% Block Ace (KAC Co. Ltd, Tokyo, Japan) and 0.25% Triton X-100 in PBS (Thermo Fisher, Waltham, MA, United States) at 4°C overnight. Rabbit anti-Beta III Tubulin primary antibodies (1:500, Abcam, Cambridge, UK) were used to confirm the presence of axonal structures in the micro-tunnels. After three rinses with D-PBS, the culture was further incubated with anti-rabbit IgG (Alexafluor 546, Thermo Fisher, Waltham, MA, United States) in 0.4% Block Ace (KAC Co. Ltd, Tokyo, Japan) and 0.25% Triton X-100 in PBS (Thermo Fisher, Waltham, MA, United States) at 4°C overnight. The culture was then rinsed with D-PBS three times. Finally, the cultures were incubated with 4′,6-diamidino-2-phenylindole (DAPI; NucBlues Fixed Cell ReadyProbes Reagent, Thermo Fisher, Waltham, MA, United States) at room temperature for 5 min to visualize cell nuclei. The samples were then examined using an inverted microscope (IX-71, Olympus, Tokyo, Japan) and a digital camera based on a CMOS sensor (C14440-20UP, Hamamatsu Photonics, Sizuoka, Japan).

### Spike Detection and Electrical Stimulation at the Electrodes

Recordings were performed after 28 days *in vitro*, after network maturation. Electrical recordings and simulations were performed using a previously developed system (Jimbo et al., 2003). The spontaneous activity of the homogeneous models was recorded for 1 h. The heterogeneous models underwent experiments in the following sequence: 30 min of spontaneous activity recording followed by 20 min of electrical stimulation.

Electrical stimulation was delivered through the electrode column furthest from the micro-tunnels in each chamber, using a previously developed system (Jimbo et al., 2003). Each electrical stimulation was a biphasic pulse with a duration of 200 μs and an amplitude of 1 V. Each stimulation epoch delivered electrical stimulation to one side of the heterogeneous coculture model followed by another electrical stimulation 10 s later to the other side, repeated 60 ×. To eliminate stimulation artifacts, data recorded within 4 ms after stimulation were excluded from the analysis. In the analysis, the first 10 s of the post-stimulation sequence after the artifact-blanking interval was considered the evoked-response activity.

**Figure 2:**
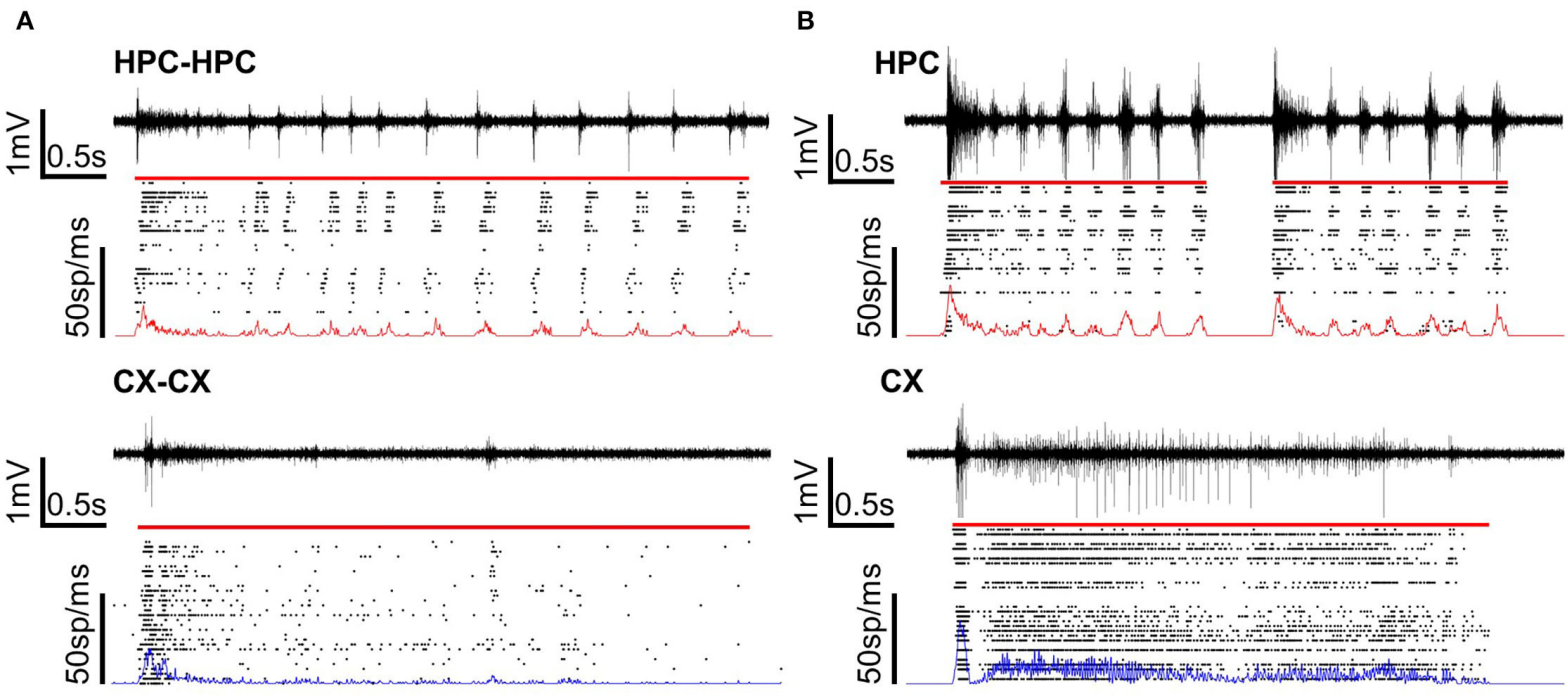
Voltage and raster plots of homogeneous and heterogeneous coculture models. **(A)** Homogeneous cocultures of CX or HPC networks. **(B)** A heterogeneous coculture of CX and HPC networks. The voltage plot shows signals recorded from one representative electrode under the sample. The raster plot shows the detected spikes over the network. Line plots on the raster plots show the firing frequency over the cultured area. The firing rate of the HPC network is in red and the firing rate of the CX network is in blue. The red line over the raster plot shows the detected length of a typical burst or burst in each type of coculture model.

Spike activities on each electrode were detected using a method based on previous studies (Takekawa et al., 2010, 2012). All raw data were processed with the Mexican hat wavelet filter to minimize noise and stimulation artifacts. Electrodes were designated as “available” by a two-step procedure. First, electrodes without noise were manually selected. Second, electrodes having a firing rate (i.e., total spike number/total recording time) greater than 0.5 s^−1^ were selected from these. Only available electrodes were used for the detection of synchronized burst and evoked-response activity. Peaks more negative than a threshold of−5 standard deviations were detected as spikes (Müller et al., 2015; Pan et al., 2015). A minimum interval of 2 ms after each spike was set to prevent repeated spike detections at a given electrode. Figure 2 shows representative activity patterns recorded from homogeneous (Figure 2A) and heterogeneous (Figure 2B) cocultures.

### Network Activity Analysis

Synchronized network burst activities were analyzed using inter-spike interval (ISI) methods and the burstiness index to quantify the characteristics of the HPC and CX networks prior to extracting the network activity properties. Synchronized network bursts were detected using slight modifications of methods from past studies (van Pelt et al., 2004; Iida et al., 2018). In brief, spike trains recorded on all available electrodes were first combined into one spike train to show the network firing rate over time. Then the network firing rate was binned with a bin width of 1 ms. In each time bin, the number of electrodes that showed firing was counted to measure the network participation rate. Then the firing rate and available electrode count in each time bin were multiplied to provide an index referred to here as the “product.” The start and end of a burst were detected by thresholding. The threshold was determined by examining the sorted product distribution. Using a rule of thumb, the threshold was defined as the height of the intersection of the sorted product distribution curve with the plot of its own radius of curvature. Lastly, the peaks in the trace of the product index greater than the threshold were detected as burst events. The start and end times of bursts were detected as the product rising above and falling below one-tenth of the threshold, respectively. A fuller explanation of the burst detection procedure is explained in Supplementary Figure 3.

The ISI is the interval between each successive pair of spikes averaged over a recorded spike train. It provides a view of spike events occurring on a single electrode, which can be independent of the synchronized network bursts. Under the circumstances of highly synchronized networks, low and high ISIs correspond to spikes occurring within and outside of bursts, respectively. To evaluate the rhythm of the firing activity occurring on all electrodes, the ISIs on all available electrodes were summed and the distribution normalized. For better visualization, the inverted ISI was calculated, and the log was transformed to provide a measure of the instantaneous frequency.

The burstiness index, first introduced by Wagenaar et al. (2005), was used as a parameter to determine the synchronicity of network firing. The firing rate was binned with time bins 100 ms wide. The burstiness index was defined as the number of spikes in the top 15th percentile of time windows by spike count/total spike number. If the network firing is tonic, the expected burstiness index is 0.15. Conversely, if the network firing is highly synchronized, the expected burstiness index is 1.

### Connectivity Analysis

To analyze the axonal connection between the networks in the two chambers, we examined the inter-network deviation of the burst start times to check if synchronized bursts occur and to identify temporal relations among burst events. To check if inter-network burst events are synchronized, we first calculated the absolute value of the start time deviation between the nearest pairs of burst events in the two chambers. Then the sequence of burst events in each chamber was shuffled and the calculation was repeated. Next, both the original deviation values and the shuffled deviation values were compiled as cumulative distribution functions to evaluate the synchronicity of the burst events. To identify temporal relations in bursts events, the ratio of bursts originating from each chamber was calculated. Here, we differentiate “chamber-initiated bursts” from “synchronized bursts” The former was identified by the difference between the start times being greater than 50 ms. To evaluate temporal relations among bursts events, the ratio of the chamber-initiated bursts originating from each chamber was calculated and compared.

In addition to analyzing burst events, we evaluated the connection between networks by cross-correlation analysis. Cross-correlation has been widely used to evaluate relationships between pairs of neurons or networks (Jimbo et al., 1999; Cadotte et al., 2008; Garofalo et al., 2009; Ullo et al., 2014; Pastore et al., 2016). To understand the relationship between cocultured networks, the cross-correlation between spike trains *S*_1_ and *S*_2_, was calculated as follows:


$$S_{x}(t)=\{\begin{array}{c} 0,\;\\ \left(if\;no\;spike\;in\;the\;bin\right)\\ 1,\;\\ \left(if\;spike\;exists\;in\;the\;bin\right) \end{array}$$

$$C\left(\tau \right)=\frac{1}{\sqrt{S_{1}^{0}S_{2}^{0}}}\sum_{t=0}^{N-\tau }S_{1}(t)S_{2}(t+\tau )$$

where *S*_1_ and *S*_2_ are the spike activities recorded from two electrodes, τ is a delay, or lag, and N is the duration of the spike train (in this case, at least 30 min). Time bin of 1 ms was used to compile the spike train. To compute the cross-correlation, the resolution of the time lag was set to 1 ms and the time frame was set to 400 ms (−400 ms to +400 ms). Next, the cross-correlation of each electrode pair was normalized by the autocorrelation of *S*_1_($S_{1}^{0}$) and $S_{2}^{0}$. Supplementary Figures 5A,B shows two typical distributions of the cross-correlation over the calculated time frame. Next, two properties were extracted from the distribution of the cross-correlations, the amplitude of the correlation peak and its position, or the tau value. The maximum value of the correlation peak can be considered the coefficient of connection strength between spike train *S*_1_ and *S*_2_, and the tau value of the correlation peak can be considered the coefficient of causality between spike trains *S*_1_ and *S*_2_. When tau > 0, there is a higher probability of spikes in *S*_1_ leading to spikes in *S*_2_; conversely, when tau < 0, there is a higher probability of spikes in *S*_2_ leading to spikes in *S*_1_.

### Evoked-Response Analysis

Inter-spike interval methods were used to analyze the evoked response. Starting from a given stimulation time to the next stimulation, 10-s epochs of network activity were evaluated. In brief, 60 evoked-response ISI distributions were averaged to permit comparison with the activity pattern recorded during spontaneous activity. The evoked activity response was detected using the thresholding method, which provides precise onset times, which is necessary to confirm activity propagation. The threshold was calculated using the production method described in the previous section. Sufficient stimulation-induced activity was defined as a product peak occurring within 500 ms after the delivery of electrical stimulation. The evoked-response rate was calculated as the number of events with sufficient activity observed in 60 trials of electrical stimulation.

## Results

### Morphological Connection Between CX and HPC

The presence of morphological connections between chambers was verified using immunofluorescence to assess the presence of axons in the tunnels. Figure 1C shows the immunostained tunnels and surrounding area. Beta III Tubulin-positive neural structures were observed within the tunnels, suggesting the entrance of axonal structures. The presence of directional axon connections was assessed by calcein-AM staining, as shown in Supplementary Figure 2. Fluorescence images of calcein-AM suggest inter-network connection from both sides of chambers in heterogeneous models.

### Connection Across Micro-Tunnels in Cocultures

Spontaneous activity was recorded from all samples to confirm the presence of activity synchronization across micro-tunnels. Across all samples, the number of available electrodes averaged 43.3 ± 7.87, suggesting that network activity was being recorded from most areas. Figure 2 shows a typical single burst in each network condition. Figure 2A shows a typical burst detected in homogeneous CX cocultures and HPC cocultures. Figure 2B shows typical bursts detected in the two chambers of heterogeneous CX-HPC cocultures. Red lines above the raster plots show the detected start and end of each burst.

**Figure 3:**
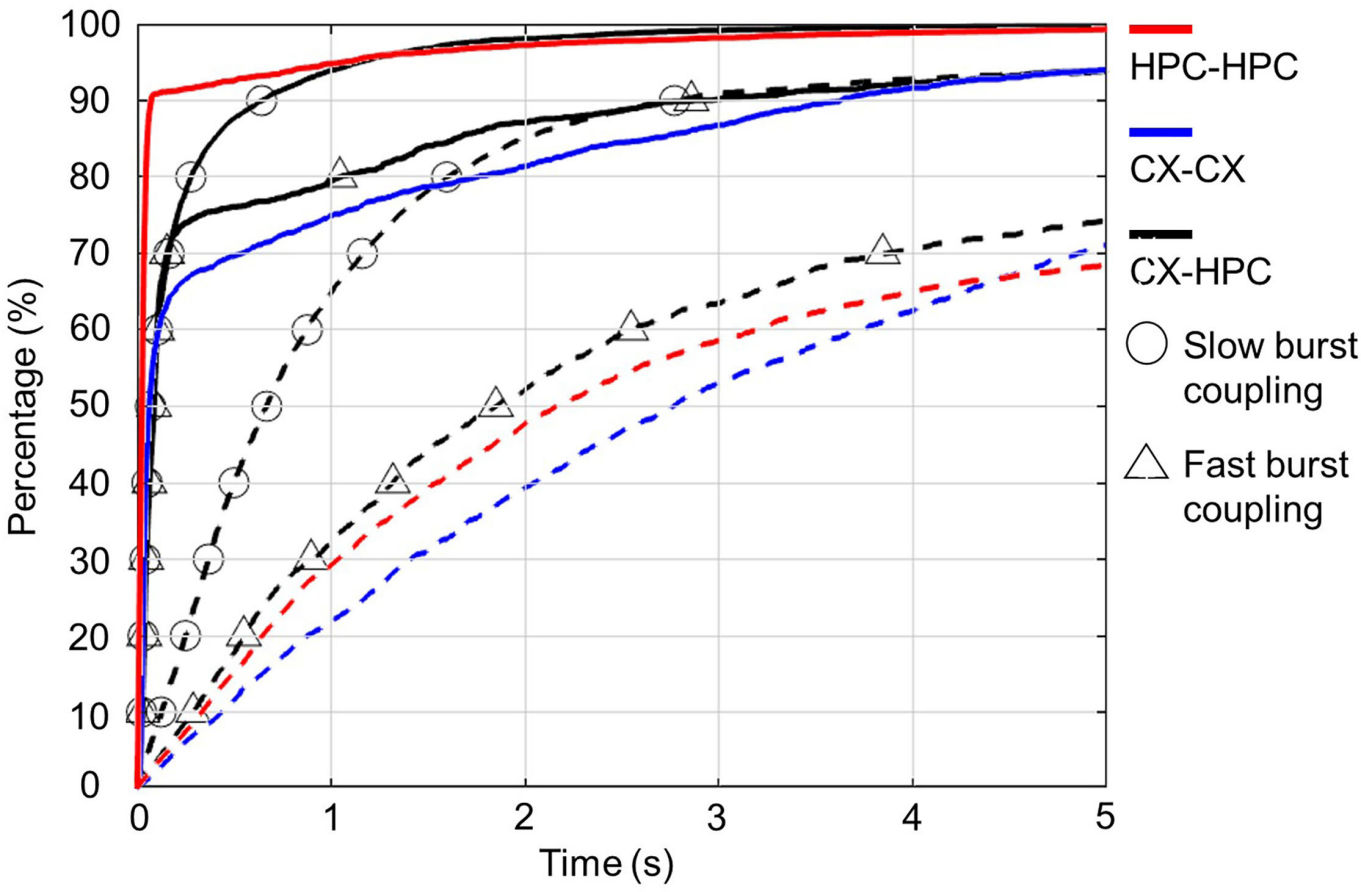
Synchronization of inter-network bursts in homogeneous and heterogeneous cocultures. Each plot shows the cumulative distribution of the absolute value of the inter-network deviation of the start times in pairs of bursts. The cumulative distribution functions (CDF) of the original start-time deviations are plotted as solid lines, and the CDFs of the shuffled start-time deviations are plotted as dashed lines. Homogeneous and heterogeneous cocultures are distinguished by color: homogenous neocortical (CX-CX) cocultures are shown in blue, and homogenous hippocampal (HPC-HPC) cocultures are shown in red, and heterogenous neocortical-hippocampal (CX-HPC) cocultures are shown in black. The CDFs of CX-HPC cocultures are further separated into two groups showing burst coupling and fast burst coupling, marked by circles and triangles, respectively. All unshuffled plots show that over 70% of the inter-network burst pairs had start-time deviations shorter than 500 ms. In contrast, the inter-network burst events show longer deviations after start-time shuffling.

To confirm activity synchronization across chambers, the average deviation between the start times of closest pairs of burst events spanning the two chambers was calculated and compared with the calculated deviation after shuffling the start-time sequence. Figure 3 shows the cumulative distribution function of the calculated start-time deviations and those of the shuffle controls, from both homogeneous and heterogeneous cocultures. The relatively high percentage of short start-time deviations suggests synchronized activity in the coculture samples. These results suggest that the networks formed a functional connection through axons that traversed the micro-tunnels.

Next, we investigated the existence of directional propagation in synchronized burst events. To investigate directional propagation, we calculated the ratio of chamber-initiated bursts originating from each chamber. The ratio of chamber-initiated bursts (start-time difference larger than 50 ms) in the control, homogeneous models, namely CX-CX and HPC-HPC, was 0.59 ± 0.22 and 0.16 ± 0.11, respectively. The corresponding figure was 0.68 ± 0.26 in the heterogeneous CX-HPC coculture model. The ratio of chamber-initiated bursts originating from the two sides in the control models was: CX-CX (chamber A initiated: 0.28 ± 0.11, chamber B initiated: 0.31 ± 0.14); and HPC-HPC (Chamber A initiated: 0.079 ± 0.064, chamber B: 0.085 ± 0.065), where chamber A and B stand for the two sides of the chamber in control models. No significant difference was shown between the ratio of chamber A and B (Two sample *T*-Test, *p* > 0.05). In the heterogeneous coculture models, the ratio of HPC-initiated bursts was significantly higher than the ratio of CX-initiated bursts (CX-initiated, 0.18 ± 0.13; HPC-initiated, 0.51 ± 0.25, Two-sample *T*-Test, *p* < 0.0001). A scatterplot of all samples is shown in Supplementary Figure 4. The results suggest a greater percentage of HPC-initiated bursts in the CX-HPC models, while the homogeneous cocultures showed lesser directional effects.

**Figure 4:**
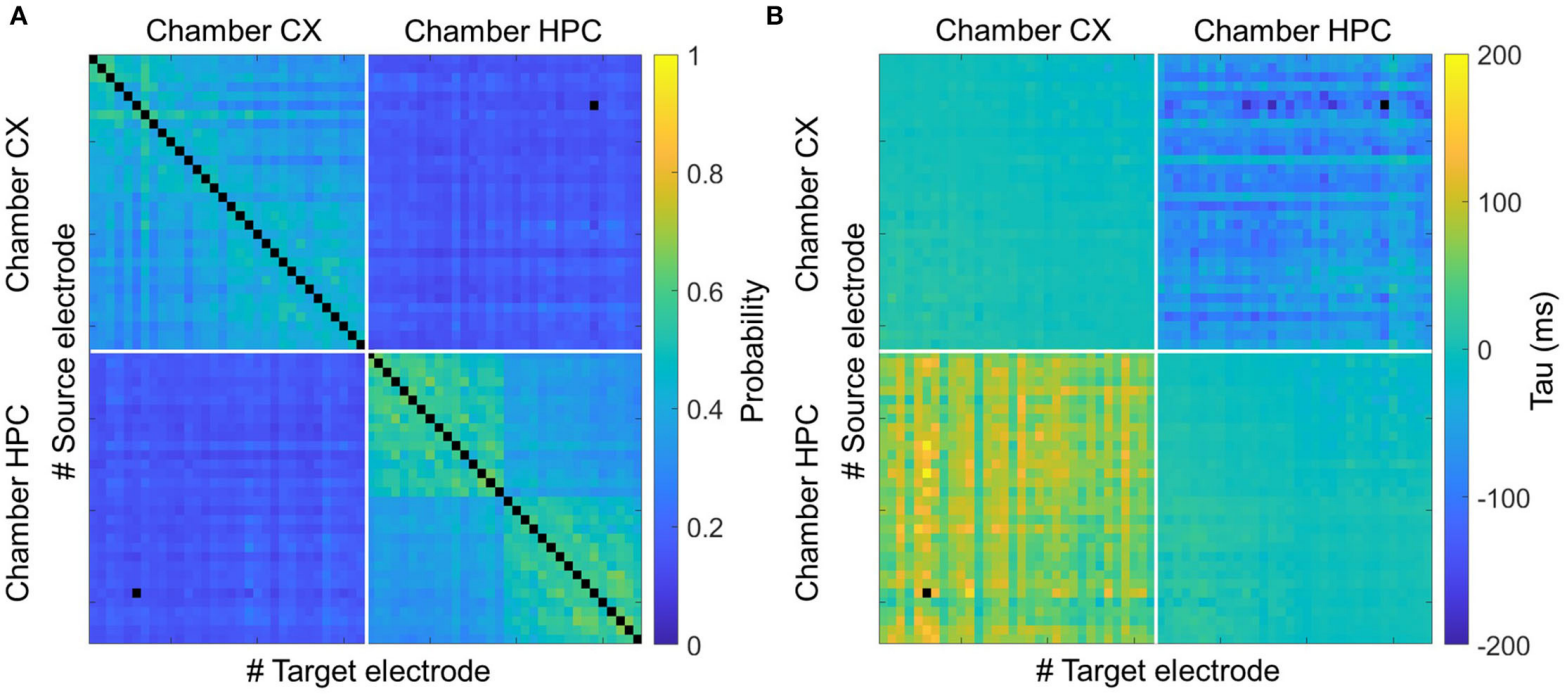
Connectivity analysis of heterogeneous coculture models by cross-correlation. **(A)** Shows a heatmap of the averaged correlation amplitude (left) and **(B)** shows a heatmap of the correlation time lags (right) for pairs of spikes occurring in heterogeneous coculture models. The CX and HPC networks are each underlain by a multi-electrode array for recording. Each matrix cell represents the correlation of one pair of electrodes. Each analyzed electrode can be in either the CX or the HPC chamber, giving four types of correlation: chamber CX-to-CX (upper left), CX-to-HPC (upper right), HPC-to-CX (lower left), and HPC-to-HPC (lower right). The amplitude matrix shows the effect of the micro-tunnel barrier in reducing interactions between networks, and the time-lag matrix shows that spikes in the HPC network lead to those in the CX network.

To confirm these results, we also assessed the connectivity between chambers by cross-correlation analysis. Supplementary Figure 5 shows two typical cross-correlation distributions between pairs of electrodes. Supplementary Figure 5A shows the distribution of the cross-correlations of pairs of electrodes within the same chamber. Supplementary Figure 5B shows the same for the case of one electrode in the CX chamber and the other in the HPC chamber. The time bin of each bar in the cross-correlation histograms is 10 ms. For clarity, hereinafter, the maximum value is referred to as the amplitude, and the tau value is referred to as the time lag. Figures 4A,B shows the average amplitude and time-lag matrices for the heterogeneous models. The data are sorted by causation as follows: upper-left section, CX-to-CX; upper right, CX-to-HPC; lower left, HPC-to-CX; and lower right, HPC-to-HPC. Supplementary Figures 5C,D shows the same in the homogeneous coculture models. The averaged amplitude and time lag of the electrodes were used to characterize these sections and were compared between samples. For amplitude, within the same chamber, the mean value is 0.39 ± 0.09 in the CX-to-CX section and 0.43 ± 0.09 in the HPC-to-HPC section. Across chambers, the mean value is 0.15 ± 0.08 (CX-to-HPC and HPC-to-CX are the same due to the symmetry of the cross-correlation formula). For time lag, within the same chamber, the mean value is 0.39 ± 0.19 ms in the CX-to-CX section and 0.44 ± 0.21 ms in the HPC-to-HPC section. Across chambers, the mean value is −66.66 ± 76.85 ms in the CX-HPC sections. Consistent with the chamber-initiated burst results, cross-correlation analysis suggests a temporal relation of HPC spikes firing occurring in advance of CX spikes (Kruskal–Wallis test, *P* < 0.0001). Meanwhile, a drop in amplitude in the CX-to-HPC and HPC-to-CX sections suggest a direct interference in cross-chamber communication by the physical barrier represented by the micro-tunnel array (Kruskal–Wallis test, *P* < 0.0001).

### Heterogeneous Cocultures Represent the Activity Rhythms of Homogeneous Cocultures

After connectivity analysis in homogeneous and heterogeneous models, we tested whether the CX-HPC (heterogeneous) coculture models were able to duplicate the activity patterns observed in the homogeneous, control cocultures (CX-CX or HPC-HPC). For elucidating network properties, we focused on instantaneous frequency (inverted ISIs) which represents rhythmic activity in the network. High-frequency components (short ISIs) refer to the spike activity within each burst, while low-frequency components refer to the intervals between bursts.

As a control, we first calculated the frequency distribution of the homogeneous cocultures. To exclude noise from the heatmaps, a lower limit of 0.002 amplitude threshold was applied to our data. CX cultures demonstrated a major peak around 20–40 Hz, which is considered low-gamma–like activity, and a minor peak between 0.1 Hz and 1 Hz, which is considered a slow-wave–like a rhythm. These data are shown in Supplementary Figure 6A. HPC cultures demonstrated two peaks: one at approximately 4–10 Hz (theta-wave–like activity) and the other at approximately 100 Hz (high-gamma–like activity), as shown in Supplementary Figure 6B. For clarity, these peaks are labeled with their frequency-band names.

**Figure 5:**
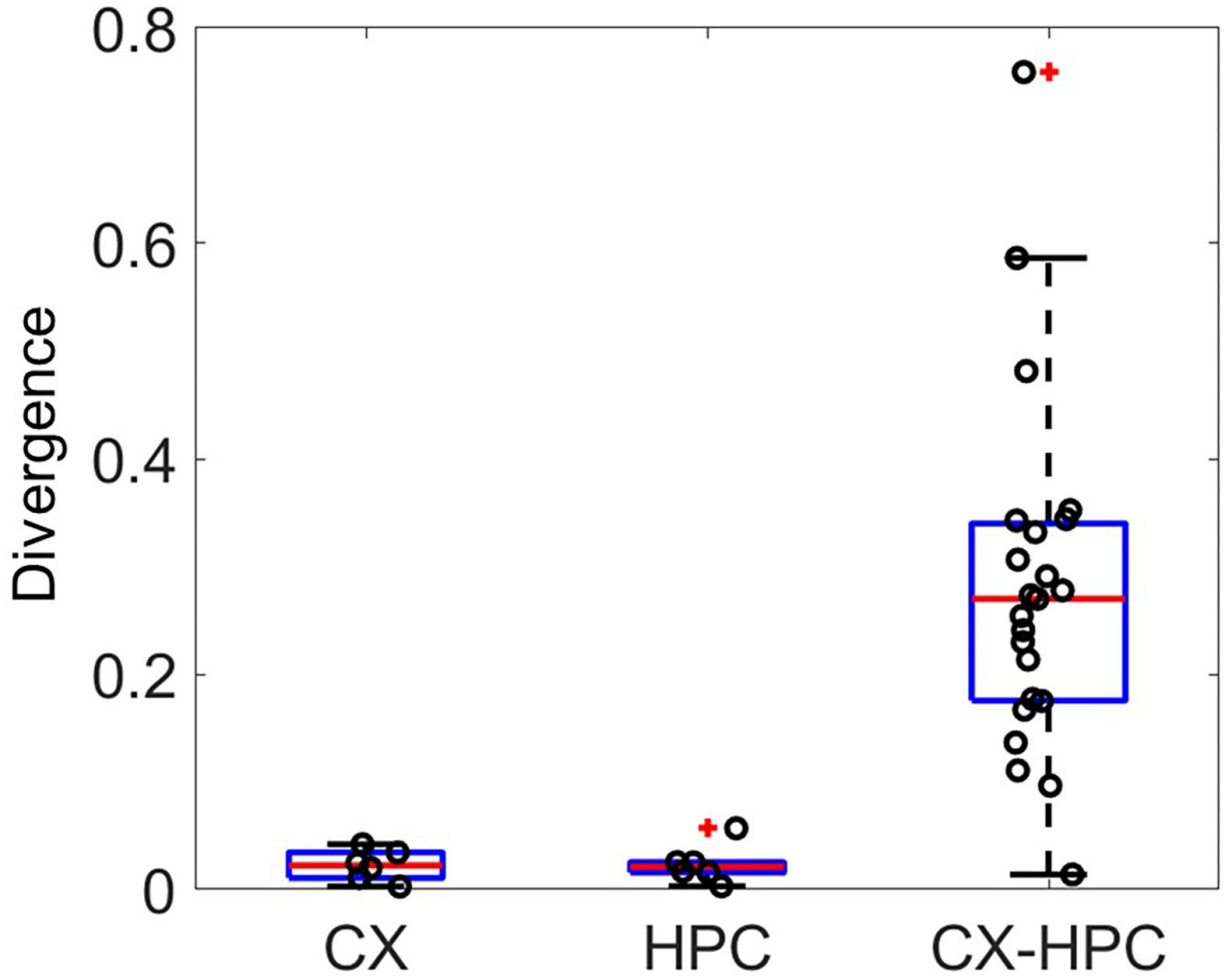
The Kullback–Leibler divergence (KLD) of the distribution of inter-spike intervals between electrodes in different culture chambers. The KLD in homogeneous cocultures of CX and HPC neurons are lower than those in heterogeneous neocortical-hippocampal (CX-HPC) coculture samples, suggesting that activity-rhythm differences between network types are maintained in the heterogeneous cocultures.

To check whether the physiological rhythms in the activities of the two source brain structures were maintained *in vitro*, we compared the ISI distributions between the two sides of the micro-tunnels. The Kullback–Leibler divergence was used to evaluate the difference between the ISI distributions, shown in Figure 5. Both homogeneous cocultures (CX and HPC) showed lower divergence compared to heterogeneous cocultures, suggesting that differential activity rhythms were maintained in each network when separated by the micro-tunnel structure.

**Figure 6:**
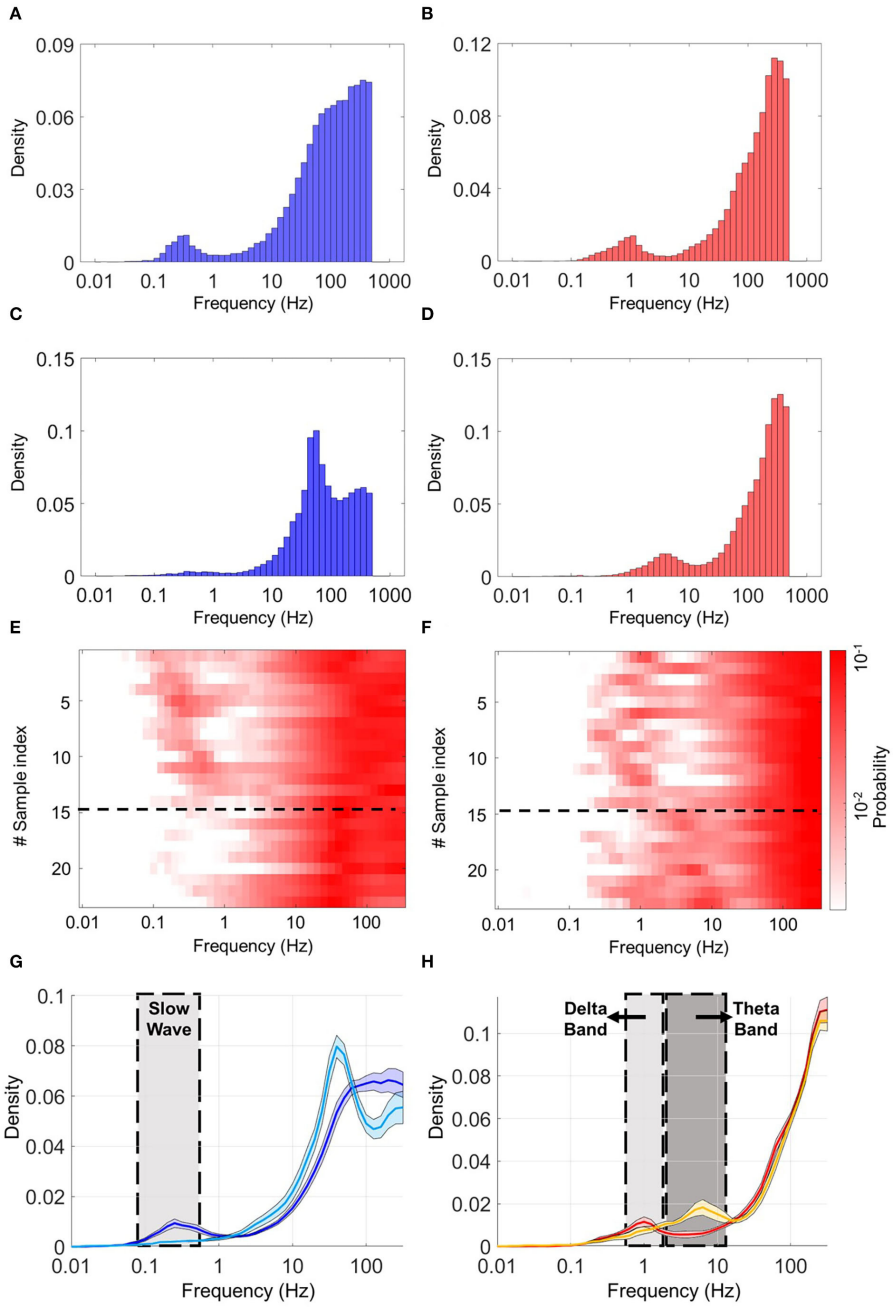
Histograms of the inverted inter-spike interval (ISI) in heterogeneous cocultures. **(A,C)** are the histograms of the CX part, **(B,D)** are the histograms of the HPC part of two typical coculture samples. **(A,B)** Show a typical sample expressing slow-burst coupling. **(C,D)** show a typical sample expressing fast-burst coupling. The CX network in **(A)** shows two peaks, in the slow-wave and gamma-frequency bands, respectively. The CX network in **(C)** shows a single peak in the low-gamma band. The HPC network in **(B)** shows two peaks in the delta and high-gamma bands, respectively. The HPC network in **(D)** shows two peaks, in the theta and high-gamma bands, respectively. **(E)** Shows a heatmap of the CX part, and (**F**) shows a heatmap of the HPC part of both samples. Corresponding rows in **(E,F)** show the CX and HPC parts of the same sample. Two patterns of frequency coupling are observed, separated by a black dotted line in the heatmaps. These are (1) slow-wave and gamma in CX, with concurrent delta and high gamma in HPC (2) and low gamma in CX, with concurrent theta and high gamma in HPC. **(G,H)** Show the average ISI distributions of the two classes of samples defined by their patterns of frequency coupling. Solid lines and shadowed areas represent the average and standard error, respectively. **(G)** Shows the average ISI distribution of neocortical spike activity. **(H)** Shows the average ISI distribution of hippocampal spike activity. Dark blue and red lines represent the first mode of activity coupling (slow-wave and gamma in CX, and delta and high gamma in HPC). Light blue and yellow lines represent the second mode of activity coupling (low gamma in CX, and theta and high gamma in HPC).

To identify differences in coupling activities in heterogeneous coculture, we first detected expression of slow-wave–like activity in the neocortical culture using an amplitude threshold of 0.005. Samples were sorted in ascending order by the expression time of slow-wave–like activity. Next, we assigned the density of the ISI distribution to a color label. In the heterogeneous coculture, two types of activity coupling were observed overall samples. Figures 6A–D shows typical ISI histograms from the two types of activity coupling in CX and HPC networks. The first type of activity coupling between HPC and CX networks was observed in 14 samples out of 23. These samples showed two peaks in the HPC and CX histograms in different frequency bands. The HPC networks are expressed at 1 Hz (delta-wave–like activity) and high-gamma–like activity (Figure 6F, upper row). The CX networks showed two peaks at approximately 0.1–0.4 Hz (slow-wave–like) and 40–100 Hz (gamma–like) (Figure 6E, upper row), a pattern referred to below as slow-burst coupling. The second type of activity coupling was observed in the remainder of the samples (9 out of 23). The peak was observed at low-gamma–like frequencies in CX networks (Figure 6E, lower row), while in HPC networks, dual peaks were observed around theta-wave–like and high-gamma–like frequencies (Figure 6F, lower row), a pattern referred to below as fast burst coupling. A sample average of the two types of activity coupling is illustrated in Figures 6G,H. The solid line shows the average, and the shadowed area shows the standard error of each distribution. These two types of activity coupling between HPC and CX may indicate the presence of different oscillation balances in the CX-HPC coculture models.

**Figure 7:**
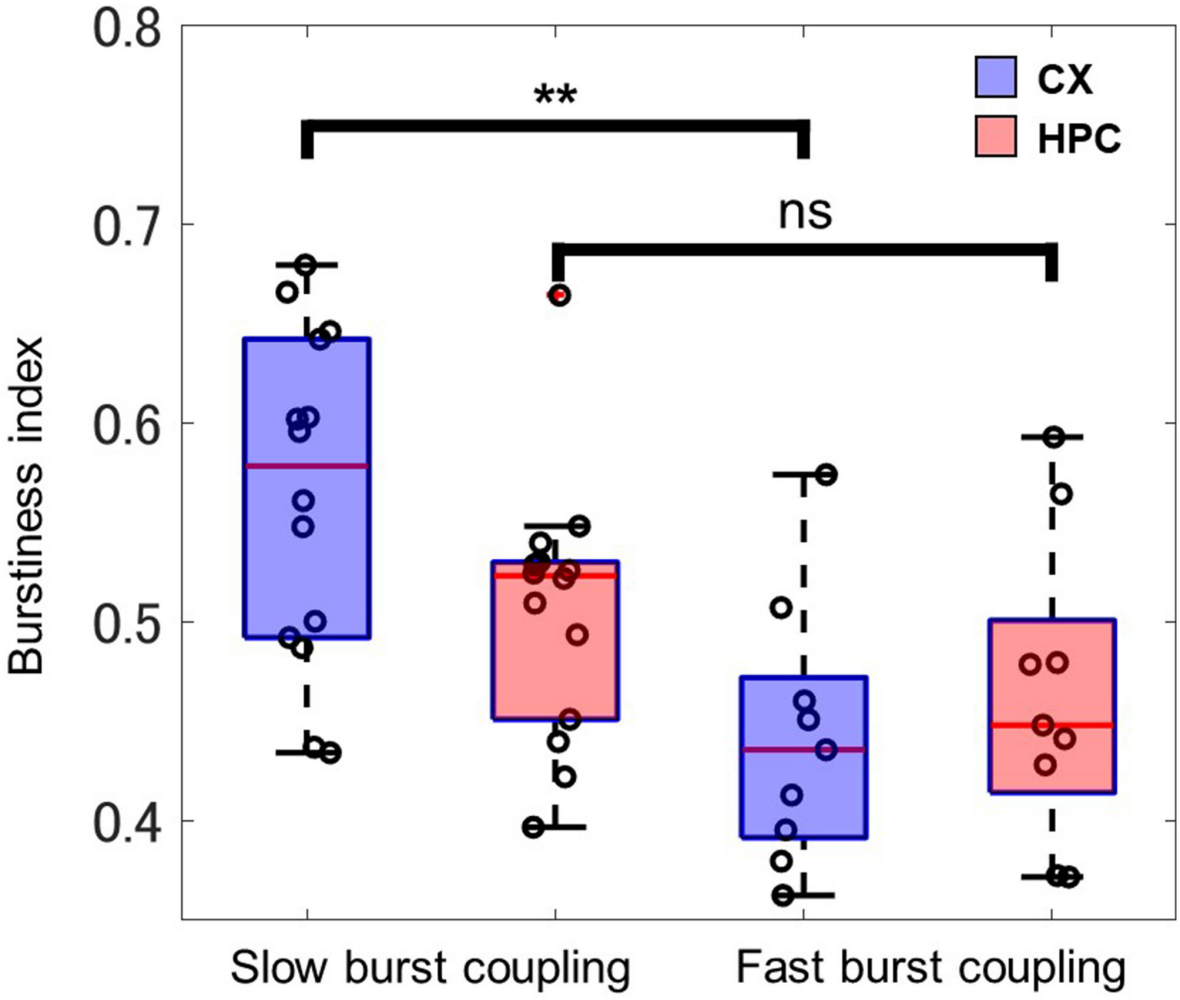
Shown is the synchronicity of firing activity in the CX and HPC part of the coculture networks, as evaluated by the burstiness index. The neocortical networks of slow-burst–coupled samples (*n* = 14) show significantly greater synchronicity (asterisks indicate a significant difference; *P* = 0.0042, Wilcoxon rank-sum) than in fast-burst–coupled samples (*n* = 9). The HPC networks show no significant difference between the slow-burst and fast-burst coupling regimes.

To evaluate activity differences in different patterns of activity coupling, the burstiness index was used to observe the synchronicity of the CX part of the heterogeneous coculture model. CX networks expressing slow-wave–like activity (14 samples, 0.56 ± 0.08) showed significantly greater burstiness (*P* = 0.0042, Wilcoxon rank-sum test) than did CX networks expressing only low-gamma–like activity (9 samples, 0.44 ± 0.07; Figure 7). No significant difference in burstiness was observed in HPC networks in either type of activity coupling. These results may indicate a difference in the information processing carried out by CX networks during the two coupling regimes.

### Evoked Response Represents Coupled Patterns of Spontaneous Activity

Our observation of activity patterns coordinated between the HPC and CX networks showed the possibility of CX-HPC coupling in which bursts share a similar start time but maintain different activity rhythms in the two networks. To establish that these paired activity rhythms represent different modes of coupling between the CX and HPC networks (slow-wave–like with delta-wave–like and low-gamma–like with theta-wave–like), single-pulse electrical stimulation was used to evoke activity within one chamber while the response in the other chamber was recorded. This was done rather than passively relying on observations of networks possessing different rhythms but sharing similar burst start times. If frequency coupling between two distinct networks exists, triggering network activity in one network should evoke a paired activity pattern in the other. For example, a CX network expressing a low-gamma–like activity should entrain a theta-wave–like a burst activity in the HPC network, and the CX network should be similarly entrained by the HPC network.

**Figure 8:**
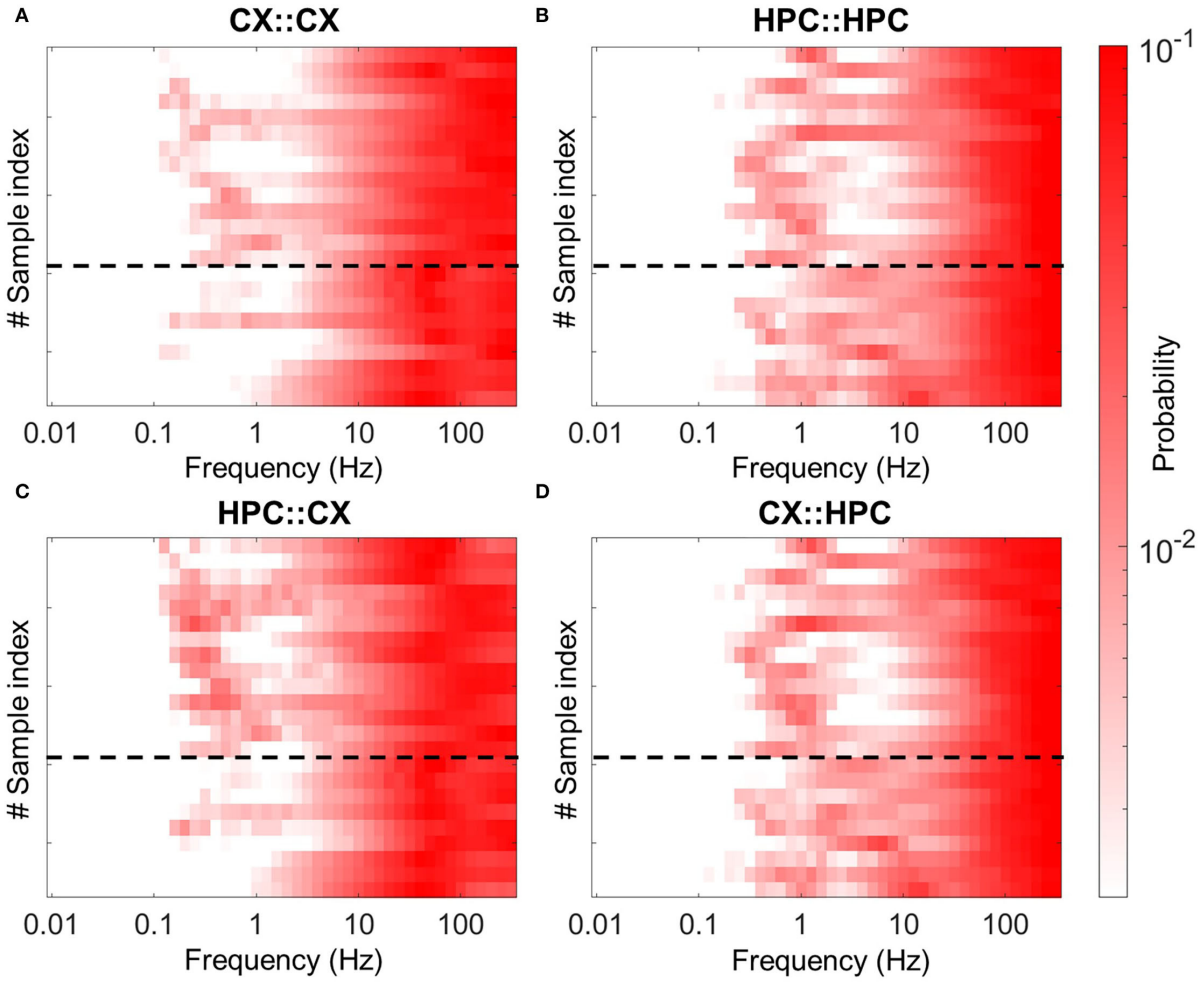
Inverted ISI heatmap of evoked responses in heterogeneous cocultures. Samples expressing different modes of activity coupling are separated by the black dotted line. Samples that express the first mode of coupling (CX, 20–40 Hz; HPC, 4–10 Hz; lower part of heatmap) show low-gamma–like activity after either CX network stimulation **(A)** or HPC network stimulation **(C)**. Consistently, the HPC network in samples with the same coupling mode express theta-wave–like activity in response to either HPC network stimulation **(B)** or CX network stimulation **(D)**. In contrast, samples that express the second coupling mode (CX, 0.1–0.5 Hz; HPC, 1 Hz) show an activity rhythm similar to that seen in spontaneous activity.

First, we verified that electrical stimulation evokes activity patterns similar to those observed in spontaneous activity. To evaluate the coupling between dissimilar networks, the ISI distribution was calculated from data recorded under four conditions. We expressed the activity pattern resulting from the evoked stimulation of X acting on Y as X::Y. Thus, the direct response of a CX network to stimulation in the CX network is expressed as CX::CX, whereas the response of an HPC network to stimulation in a CX network is represented as CX::HPC. Consistent with the distribution of spontaneous activity, the ISI pattern showed similar distributions in CX::CX and HPC::HPC, with the two types of patterns found in similar sample sets (Figures 8A,B). Slow-burst–coupled samples showed two peaks in the ISI distribution of the evoked response in slow-wave–like and gamma-frequency bands in the CX::CX condition, and in delta-wave–like and high-gamma bands in HPC::HPC (Figures 8A,B, upper rows). Likewise, the ISI distribution of the evoked response in fast-burst–coupled samples showed low-gamma band activity in the CX::CX condition, and theta and high-gamma band activity in the HPC::HPC condition (Figures 8A,B, lower rows).

Next, we checked if the evoked response in the counterpart was able to show a paired coupling pattern. Consistent with the distribution of the spontaneous activity, slow-burst–coupled samples showed paired coupling activity at delta-wave–like and high-gamma bands in the CX::HPC evoked response, and in slow-wave–like and gamma bands in the HPC::CX evoked response (Figures 8C,D, upper rows). Likewise, fast-burst–coupled samples showed theta and high-gamma activity in the CX::HPC evoked response, and low-gamma band activity in the HPC::CX evoked response (Figures 8C,D, lower rows). These results suggest that electrical stimulation was able to trigger paired, coupled activity in a heterogeneous coculture model. The average ISI distribution in the four conditions of the evoked response is shown in Supplementary Figure 7.

**Figure 9:**
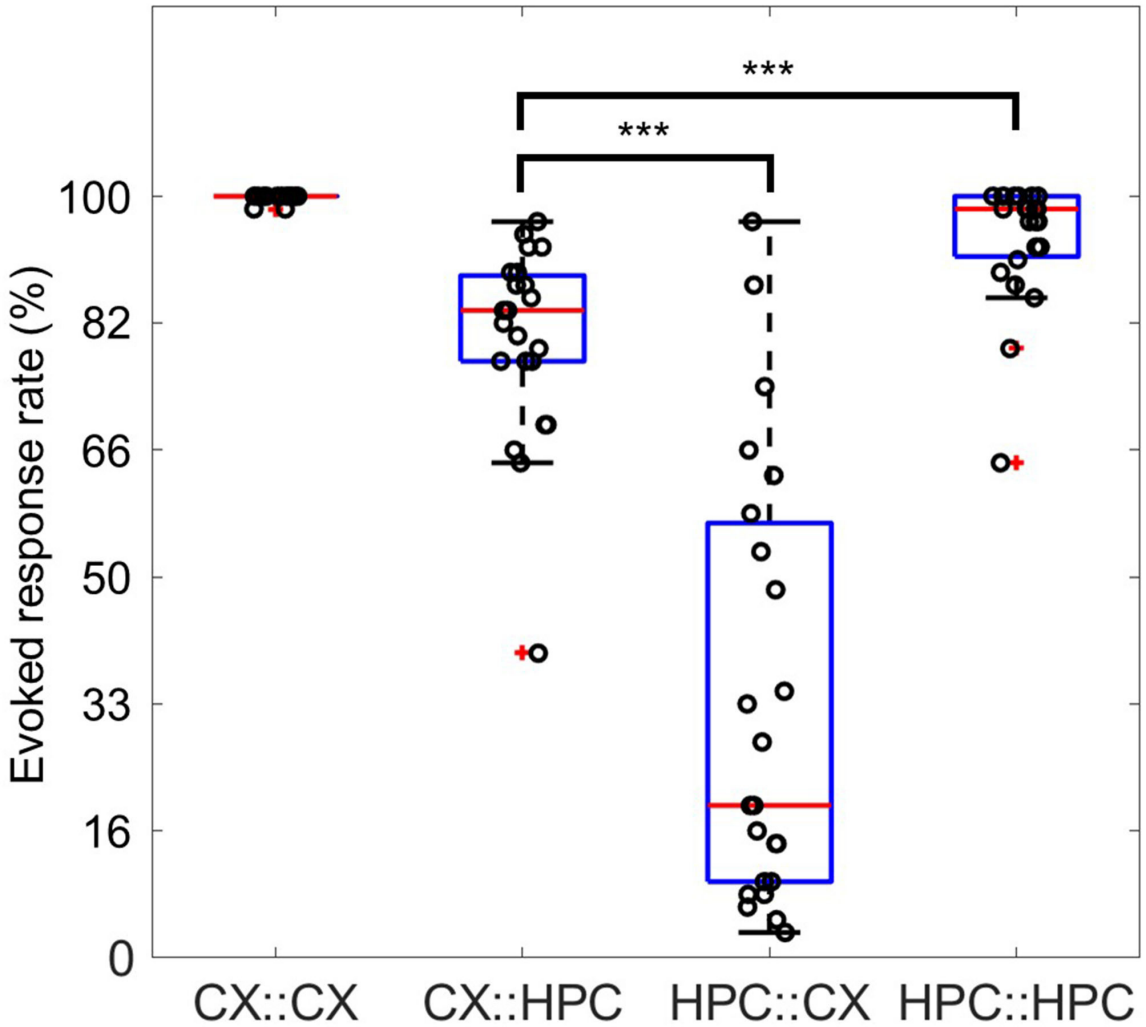
Response rates for direct network stimulation and cross-chamber evoked responses. Four possibilities for evoking a response are illustrated. The response rate of X to stimulation in Y is expressed as Y::X. The direct response of the CX network to stimulation in the CX network is expressed as CX::CX, whereas the response of the HPC network to stimulation in the CX network is expressed as CX::HPC. Each data point displays the rate of observation of evoked responses possessing sufficient firing activity within 500 ms after stimulation. The direct evoked response in both CX::CX (0.9986 ± 0.0048) and HPC::HPC (0.9435 ± 0.0828) showed a response rate >90%. The cross-chamber response CX::HPC (0.8123 ± 0.1263) shows a rate around 80%. However, over half of the samples show a response rate lower than 50% in the reverse direction, HPC::CX. The direct evoked response in HPC::HPC is significantly greater than the cross-chamber response in CX::HPC (***, *P* < 0.0001, Mann–Whitney U-test). The cross-chamber evoked response in CX::HPC is significantly greater than the cross-chamber response in HPC::CX (***, *P* < 0.0001, Mann–Whitney U-test).

It should be noted that during the confirmation of activity propagation across tunnels, we found significantly different response rates within 500 ms of stimulation in the CX::HPC and HPC::CX pathways. Response rates within 500 ms were calculated by detecting whether sufficient network firing occurred within 500 ms of stimulation in either direction. The cross-chamber evoked responses showed a significantly greater network firing in CX::HPC than in HPC::CX (*P* < 0.0001, Mann–Whitney U-test) (Figure 9). There was no significant difference (*P* = 0.1302, Mann–Whitney U-test) between the response rates of slow-burst– and fast-burst–coupled samples (*n* = 14 and 9, respectively).

To this point, we have shown that by photolithographic techniques, we could create micro-tunnel structures able to maintain separate activity rhythms in individual networks but allow them to communicate with each other. Analysis of burst start times and cross-correlations revealed a temporal relation between HPC and CX networks, with HPC spike firing occurring in advance of CX spikes. In terms of instantaneous frequency, we showed that two types of frequency coupling between CX and HPC were present in both spontaneous and evoked activity. The two types of coupling showed different levels of synchronicity in the CX networks. Subsequently, we showed that an evoked response due to electrical stimulation in one network was able to trigger a paired firing pattern in the other. Finally, the early response to electrical stimulation showed a significantly higher response rate in the CX::HPC pathway than in the HPC::CX pathway.

## Discussion

We established a coculture model comprising two different but interacting networks derived from, respectively, the embryonic rat HPC and CX. The coculture models were able to maintain the dissimilar activity rhythms of the individual networks while allowing communication between these heterogeneous networks. Analysis of burst start times and cross-correlations suggest that HPC spikes lead to CX spikes. Activity recorded during spontaneous activity and evoked responses showed two types of activity coupling between the HPC and CX networks, by which the coculture samples could be separated into two groups. The first group showed slow coupling of burst activities, with the HPC network expressing a burst activity rhythm at 1 Hz while the CX network expressed a burst activity rhythm at 0.1–0.4 Hz. The second group showed fast coupling of burst activities, with the HPC network expressing a burst activity rhythm at 4–10 Hz while the CX network expressed a firing activity at 20–40 Hz. Both types of CX burst activities were observed in control CX models. HPC networks expressing an activity rhythm at 4–10 Hz were also observed in control HPC models. In the CX network, the two types of activity coupling showed different levels of firing synchronicity. Despite the difference in burst rhythm and firing synchronicity level, both groups showed an asymmetrical response rate in the cross-chamber early evoked response, expressing significantly higher firing rates in the CX::HPC pathway.

Hippocampal control models showed a burst activity rhythm at 4–10 Hz. Such an activity rhythm has also been reported by other studies on *in vitro* HPC monocultures (Charlesworth et al., 2015; Gladkov et al., 2018) and slices (Goutagny et al., 2009). Contrastingly, CX control models showed periods of concentrated firing activity, which were separated by quiescent periods of low activity. The firing-quiescence rhythms observed in CX cultures have been reported by other researchers, and are stated to be the default firing mode in CX networks (Corner, 2008; Hinard et al., 2012; Colombi et al., 2016; Bandarabadi et al., 2020). Our homogeneous control models also exhibited the burst activity rhythm reported in monocultures, suggesting that our coculture models were able to duplicate the firing characteristics of the *in-situ* networks.

Our network analysis using the ISI method can easily be affected by sparse firing activity on each electrode. If a single firing occurs in the middle of two on-frequency firing events, the frequency detected by the ISI method will be doubled. Consequently, the observed difference between the two patterns of burst activity coupling could be due to sparse firing events occurring between synchronized bursts. However, analysis using the burstiness index showed significantly higher synchronicity in slow-burst–coupled samples than in fast-burst–coupled samples. The results of this study support the validity of network activity analysis using the ISI method.

We investigated two aspects of burst activity coupling between CX and HPC networks: (1) entrainment of the burst activity rhythm of one network by the other, and (2) expression of two coupling patterns in CX-HPC coculture models. Paired-electrode activity coupling was observed in the inter-network evoked responses going each way, and results recorded during spontaneous activity showed two types of burst activity coupling.

Concerning the first aspect, it has been reported that the properties of the evoked response resemble those observed in spontaneous activity (Luczak and MacLean, 2012; Pasquale et al., 2017). Moreover, stimulation of specific subsets of the network and stimulation of specific leading neurons evoked similar network burst activity (Eytan and Marom, 2006). The above characteristics support the similarity of the evoked response in stimulated networks to the recorded spontaneous activity. Considering this, the coupled activity observed in the counterpart of the stimulated network supports the existence of two coupling patterns in the developed CX-HPC coculture model.

Second, in control models, CX and HPC networks showed different burst activity rhythms. The heterogeneous coculture models, consisting of two subnetworks generating different firing activity rhythms, may express a self-organized activity-rhythm dependent on the rhythms of the two subnetworks. Networks under the first type of CX-HPC coupling (CX: 0.1–0.4 Hz, HPC: 1 Hz) could resemble the stable state observed in control models, while those under the second type (CX: 20–40 Hz, HPC: 4–10 Hz) could be another dynamically balanced point between CX and HPC networks.

In connectivity analysis, intriguingly, we found a higher ratio of burst events originating with HPC bursts, and HPC spike events consistently led to CX spike events, suggesting a leading role of HPC firing activities in heterogeneous cocultures. However, using electrical stimulation, we demonstrated a higher evoked-response rate in the CX::HPC pathway than in the HPC::CX pathway, suggesting an opposite direction of activity propagation in heterogeneous cocultures. Two factors were considered to explain the phenomenon. The first is the difference in HPC and CX membrane dynamics at the cellular level. HPC networks have shown faster burst rhythms than CX networks. Synchronized burst events in the HPC network can be interrupted by pharmacologically blocking the persistent sodium channel (Penn et al., 2016). Persistent sodium channels can raise the subthreshold membrane potential, resulting in greater excitability of the HPC neurons. Greater excitability of the HPC neuron and its network may explain the temporal relation between HPC and CX network bursts. Meanwhile, the different excitabilities of the CX and HPC networks can lead to different rates of response to excitation delivered by an axonal connection through the micro-tunnels.

The second factor is the nonsymmetrical excitatory/inhibitory input between CX and HPC reported by Brofiga et al. (2021). In that study, coculture models showed an inhibitory input from the HPC to the CX network. In contrast, CX networks established a larger number of intra-network connections (Brofiga et al., 2021). This nonsymmetrical inter-network communication can lead to a pronounced recurrent network activity circulating between the HPC and CX subnetworks, with the CX network acting as an oscillation generator and the HPC acting as a regulator to control the overall rhythm.

Furthermore, throughout our observation time (spontaneous activity, 30 min; evoked response, 20 min), each sample showed one major type of activity coupling between the CX and HPC networks, with a small admixture of the other component. Two explanations for this can be considered. First, the synchronized firing activity of a mature neuronal network can represent a stable state (Rolston et al., 2007; Slomowitz et al., 2015). Because the firing activity depends on the structures of both the inter- and intra-network connections, a stabilized structure may only express one pattern of coupling. Second, the two patterns of activity coupling may represent two dynamically balanced points of a larger recurrent network, which switches between balance points with a period longer than our observation time. Nevertheless, both conditions imply the presence of an unexpressed burst activity coupling pattern in addition to the observed coupling pattern.

The observed dependence of the synchronicity level on the pattern of burst activity coupling implies that CX-HPC coculture models have different information processing properties under different coupling regimes. Understanding these differences can provide insights into how information is processed as the coupling state varies. However, because the current study focused on analyzing burst activity, which accounted for most of the observed spikes, it should be noted that a separate analysis of spike activity outside of burst events should be performed to further elucidate the cellular dynamics of the system. Although we observed only one main pattern of burst activity coupling in any sample, CX-HPC coculture models may have the potential to express different coupling patterns over time or in response to external stimulation. Using pharmacological interventions or extreme electrical stimulation, it may be possible to shift the model between different states of activity coupling. Combined with our stimulation method, the developed model can be used to understand how shifting between coupling states can affect the transformation of information as it passes between the HPC and CX networks.

With the aim of understanding how shifting between states of cross-frequency coupling can influence memory formation, we proposed a simplified CX-HPC coculture model to simulate coupling between the HPC and CX networks. Electrophysiological recordings of spontaneous and evoked activity both revealed two types of coupling patterns. Using synchronicity analysis, we found that coculture networks that express different coupling patterns can have different information processing states. Our connectivity analysis showed that firing activity in the HPC network led to that of the CX network. Finally, the cross-chamber response rate to electrical stimulation showed a higher response rate in the CX::HPC pathway than in the opposite direction. The expression of different coupling patterns in the CX-HPC coculture model may result in a self-organized coupling between two networks oscillating under different rhythms. Our results showed the possibility of a CX-HPC coculture network possessing multiple states of activity with a different coupling mode for each. With the addition of external stimulation, the proposed CX-HPC coculture model can serve as a platform for investigating how the shifting of coupling states between CX and HPC networks *in vitro* can affect information processing.

## Data Availability Statement

The raw data supporting the conclusions of this article will be made available by the authors, without undue reservation.

## Ethics Statement

The animal study was reviewed and approved by the Ethics Committee of the University of Tokyo.

## Author Contributions

CC conceived, designed, and performed the experiments and took the lead in writing the manuscript. CC and TF performed the analysis. TA and KS verified the analytical methods and revised the manuscript. KS and KK helped supervise the project. YJ supervised the project. All authors have read and approved the final manuscript for publication. All authors agree to be accountable for all aspects of the work in ensuring that questions related to the accuracy or integrity of any part of the work are appropriately investigated and resolved.

## Funding

This work was partially supported by the Japan Society for the Promotion of Science through Grants-in-Aid for Scientific Research (KAKENHI), Grant Numbers 19H04437 and 20H04498, and a Grant-in-Aid for JSPS Fellows, Grant No. 20J22902.

## Conflict of Interest

The authors declare that the research was conducted in the absence of any commercial or financial relationships that could be construed as a potential conflict of interest.

## Publisher's Note

All claims expressed in this article are solely those of the authors and do not necessarily represent those of their affiliated organizations, or those of the publisher, the editors and the reviewers. Any product that may be evaluated in this article, or claim that may be made by its manufacturer, is not guaranteed or endorsed by the publisher.
