# Supplementary material for: Coupling of in vitro Neocortical-Hippocampal Coculture Bursts Induces Different Spike Rhythms in Individual Networks
Source: Front Neurosci. 2022 May 23;16:873664. doi: 10.3389/fnins.2022.873664 (PMC9168126; doi:10.3389/fnins.2022.873664)
Supplement: Supplementary file 1 [file Data_Sheet_1.pdf]

Supplementary Material

1 Supplementary Data

Calcein AM cell staining

The growth of axons into the micro-tunnels was observed by fluorescent staining with calcein acetoxymethyl (calcein AM; Dojindo). Calcein AM is used for observing the morphology of living cells. The acetomethoxy group enables calcein AM molecules to be transported through cell membranes and into the intracellular space. After transport, intracellular esterases remove the acetomethoxy group so that the calcein molecules are trapped inside the cell and impart to it a strong green fluorescence. Stock solution was prepared by diluting calcein AM (final concentration, 5 μM) in HEPES solution. To load calcein AM into neuronal cells, culture medium was replaced by stock solution, and the sample was incubated in an incubator for 15 min. The direction of axon entrance was investigated by loading stock solution into one chamber and only HEPES solution into the other. Concurrently, HEPES solution was warmed to 37°C for later use in rinsing the microdevice. After rinsing with warm HEPES solution 3×, the microdevice was returned to the incubator for another 15 min. This step allowed time for the hydrolysis of calcein AM to calcein and its transport into neurites. The stained samples were observed by fluorescent microscope (IX-71; Olympus) and the fluorescence images were acquired with excitation by blue light (wavelength 488 nm).

2 Supplementary Figures and Tables

2.1 Supplementary Figures

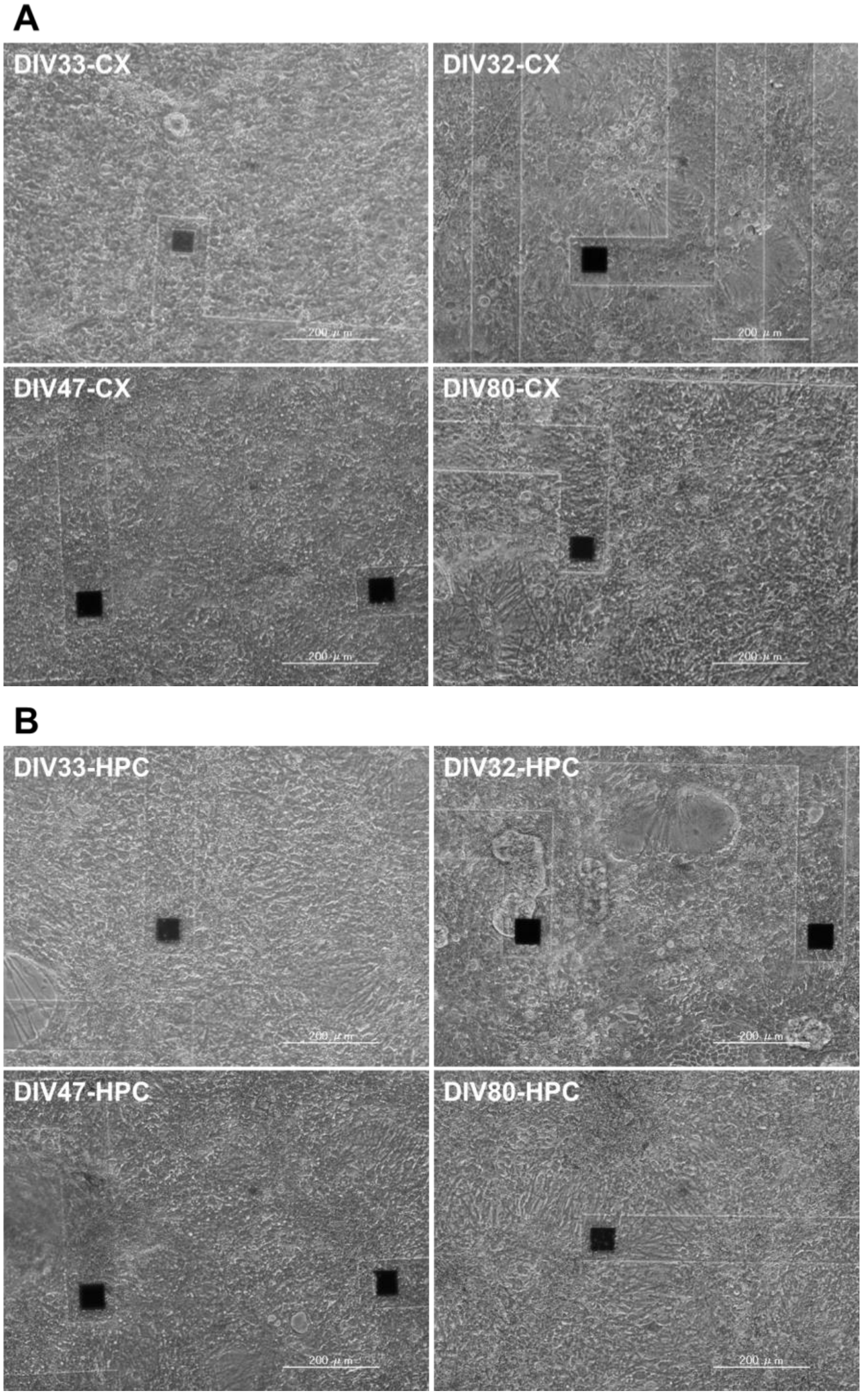

Supplementary Figure 1. Culture conditions of the neocortical and hippocampal networks. (**A**) phase contrast micrographs of cultured neocortical (CX) networks. (**B**) phase contrast micrographs of cultured hippocampal (HPC) networks. The micrographs show the condition of one sample taken on various days *in vitro* (DIV). The micrographs in (**A**) and (**B**) sharing the same DIV were taken from the same sample, while micrographs labeled with different DIVs were seeded on different days.

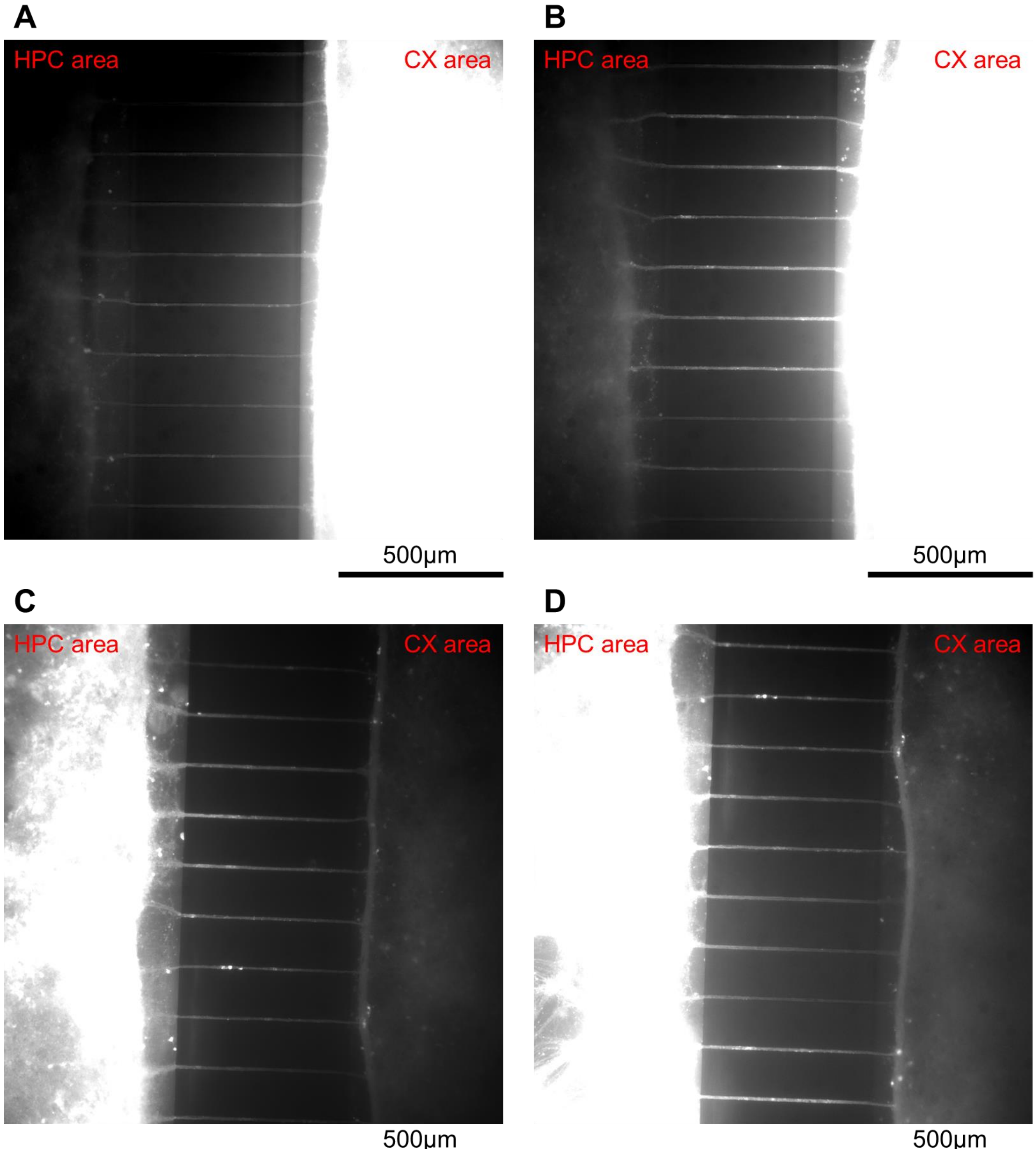

Supplementary Figure 2. Fluorescence images of heterogeneous cocultures stained by calcein AM. (**A**) and (**B**) are were acquired when calcein AM was loaded into the neocortical (CX) culture area. (**C**) and (**D**) were acquired when calcein AM was loaded into the hippocampal (HPC) culture area.

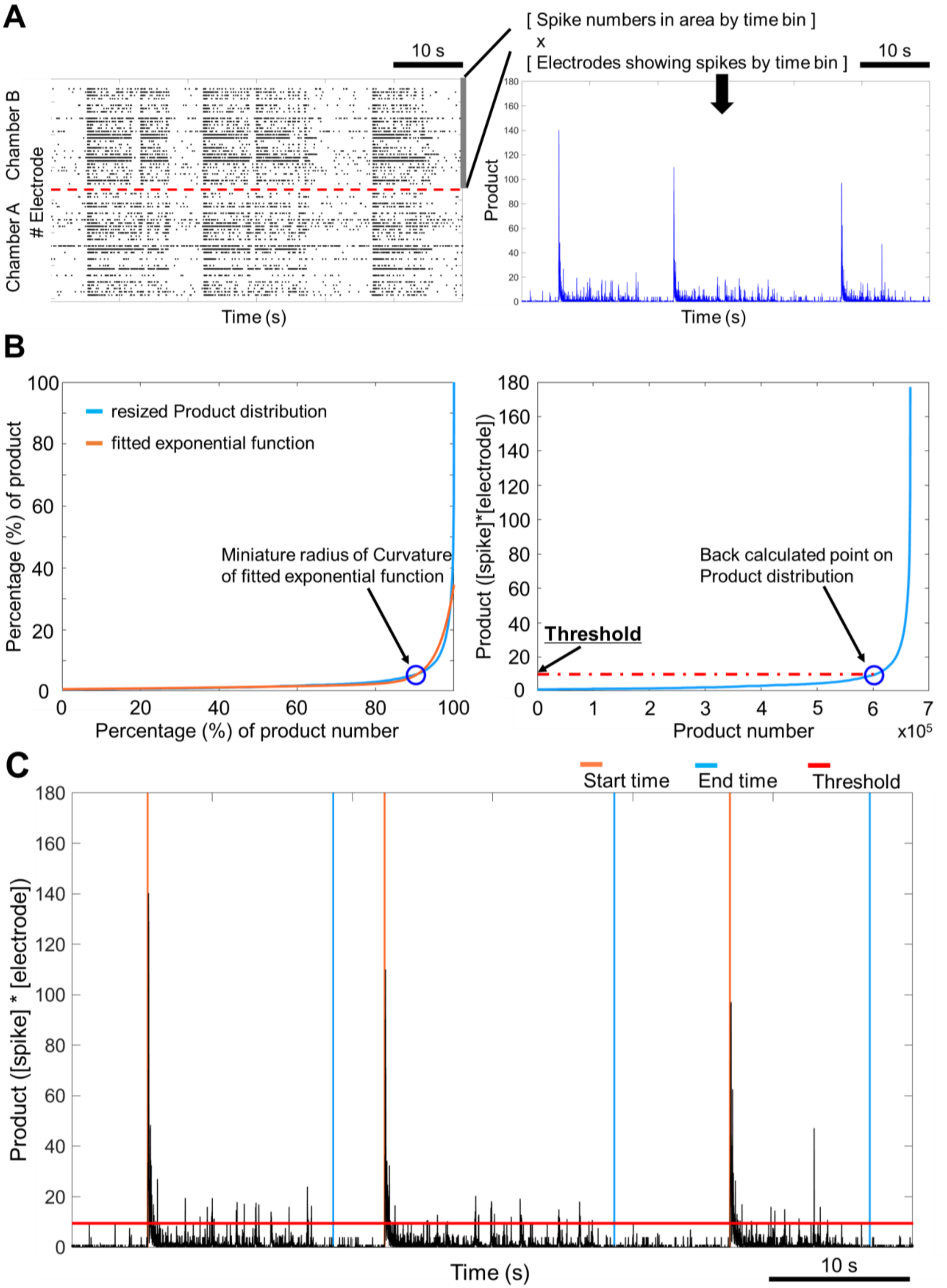
A
10 s
Chamber B
Chamber A
Electrode
Time (s)
[Spike numbers in area by time bin]
x
[Electrodes showing spikes by time bin]
10 s
Product
Time (s)
B
resized Product distribution
fitted exponential function
Percentage (%) of product
Miniature radius of Curvature
of fitted exponential function
Percentage (%) of product number
Product ([spike]*[electrode])
Threshold
Back calculated point on
Product distribution
Product number
x10⁵
C
Start time
End time
Threshold
Product ([spike] * [electrode])
Time (s)
10 s

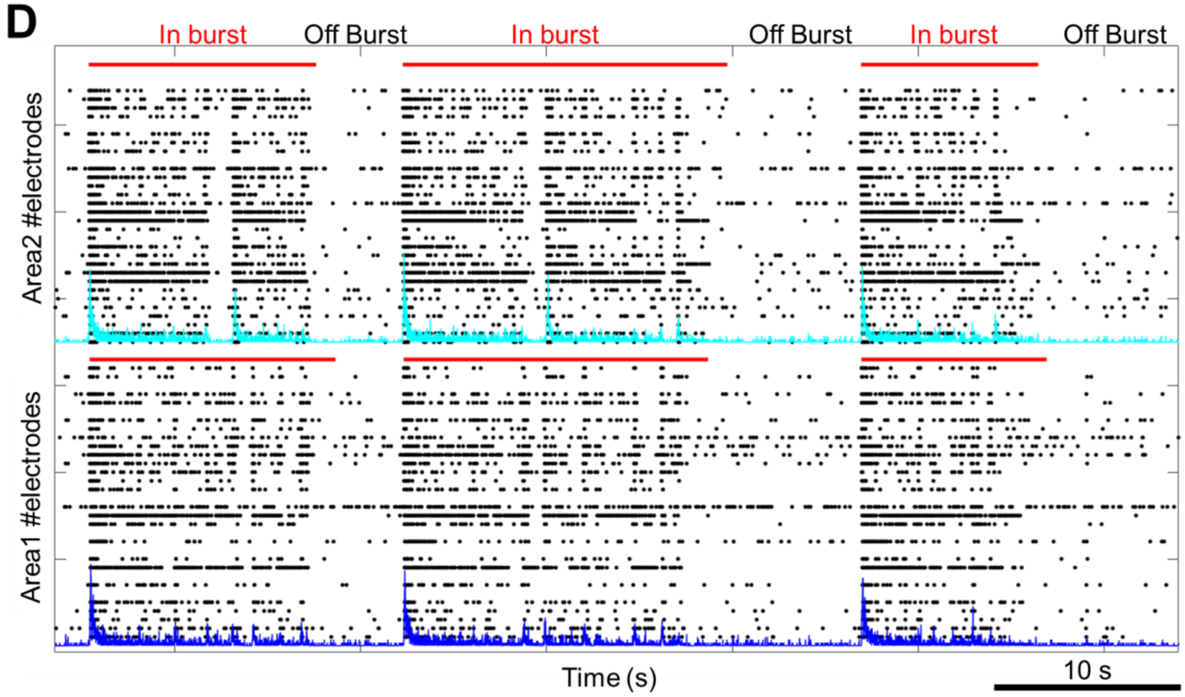

Supplementary Figure 3. Detection process for network bursts. (**A**) Calculation process of the product vector for burst detection. Left panel in (**A**) show the raster plot spike events over electrodes. Black dots show each single spike event detected on the electrodes. First, spike trains recorded on all available electrodes were combined into a single train to show the network firing rate over time. Next, the network firing rate was binned with bins of 1 ms. Meanwhile, the number of electrodes that showed firing was counted in each time bin to measure the network participation rate. Next, the firing rate and active electrode number in each time bin were multiplied to provide an index referred to as the product (**A**, right panel). (**B**) Threshold calculation for burst detection. To determine the threshold, we first sorted the calculated product in ascend order. Followed by sorting, product distribution was resized into percentage form to acquire equal length on x axis and y axis (**B**, left panel). Next, resized product distribution was fitted by exponential function. As a rule of thumb, the threshold was defined as the intersection of the miniature radius of the curvature and the fitted exponential function, and back calculated to original production distribution (**B**, right panel). Threshold is determined as the y value of back calculated point on the product distribution. (**C**) Start and end time detection of bursts. The peaks in the product trace higher than threshold were detected as burst events. The start and end times of bursts were detected as the times at which the product rose above or fell below one-tenth of the threshold, respectively, indicated by orange and blue vertical lines. Lastly, bursts with inter-burst interval (time interval of current burst start time and previous burst end time) shorter than 500 ms were combined, considered as the same burst event. (**D**) shows burst events detected from a coculture sample. Blue and cyan lines on raster plot show the network firing rate in each chamber. Red lines above raster plots show the start and end time of each detected burst event.

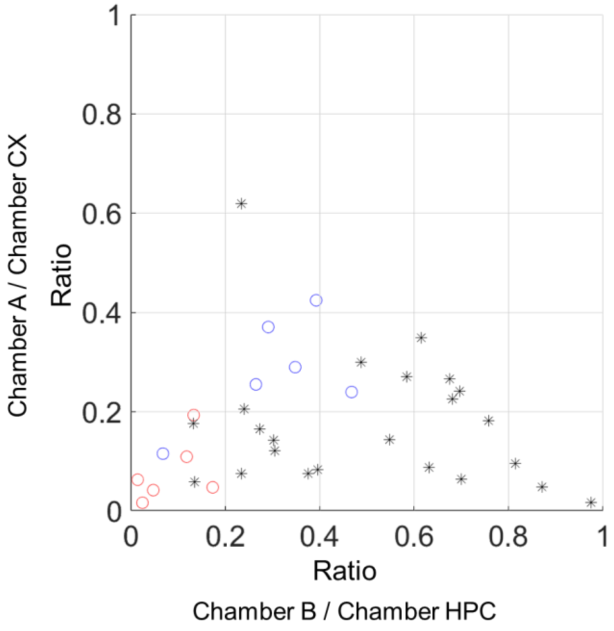

Supplementary Figure 4. Scatterplot of chamber-initiated bursts in the coculture models. Data points of hippocampal (HPC) and neocortical (CX) homogeneous cocultures are presented as red and blue circles, respectively. Heterogeneous cocultures are presented as black asterisks. Chamber A and B labels next to the X and Y axes represent the two chambers in homogenous cocultures. Chamber HPC and Chamber CX, on the other hand, represent the ratio of chamber-initiated bursts originating from HPC and CX, respectively. Most data points from heterogeneous cocultures have a high ratio of HPC-initiated bursts to CX-initiated bursts, which indicates a higher probability of the HPC burst leading the CX burst than the reverse. In contrast, homogeneous cocultures show a lesser tendency for inter-chamber burst initiation.

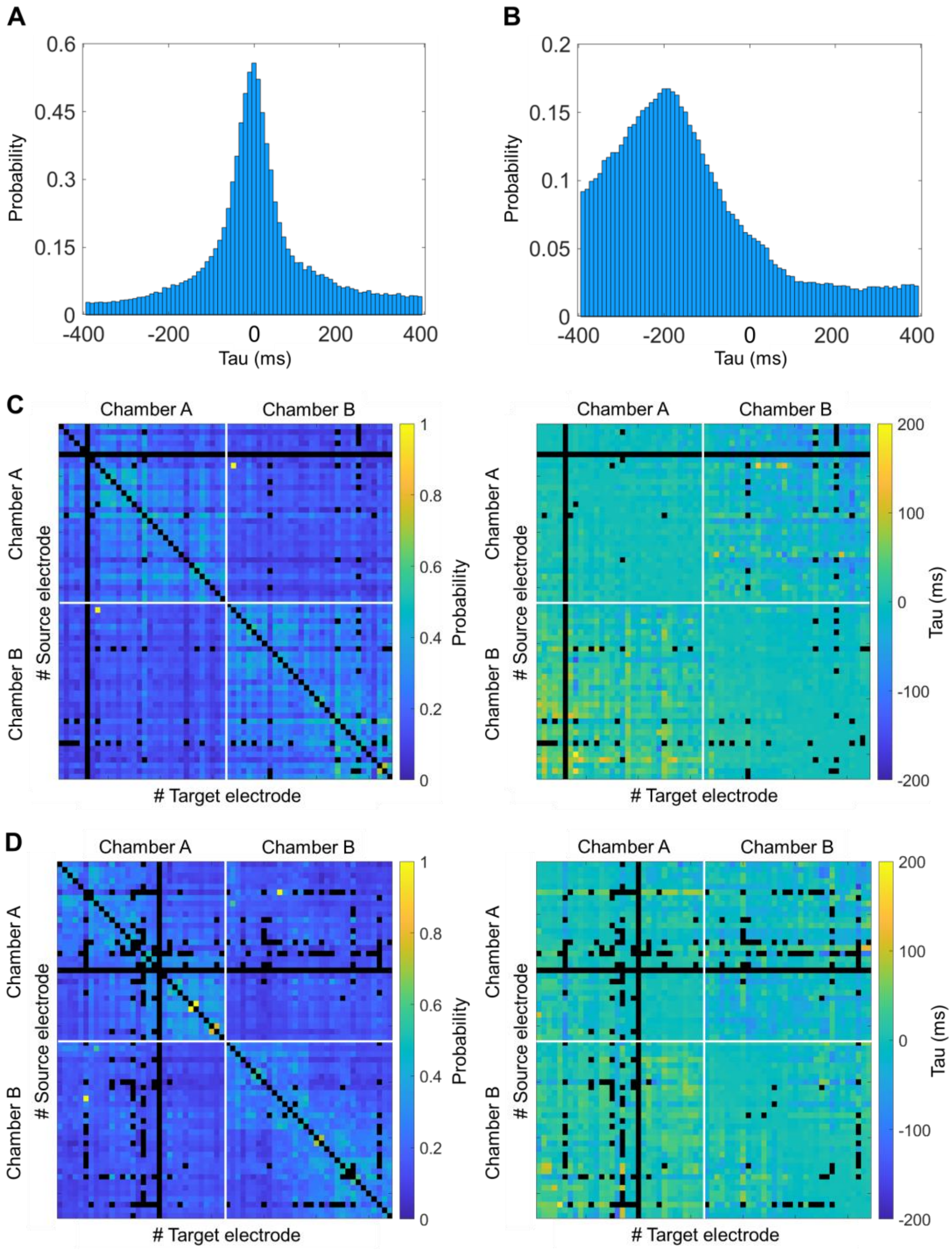

Supplementary Figure 5. Analysis of the inter-chamber connections by cross-correlation. (**A**) and (**B**) are typical cross-correlation histograms of pairs of electrodes within the same chamber and in different chambers, respectively. (**C**) and (**D**) show the averaged amplitude (left panel) and time lag (right panel) of homogeneous coculture models. The data are sorted by causation as follows: upper-left section, A to A; upper right, A to B; lower left, B to A; and lower right, B to B. The averaged amplitude and time lag of the electrodes were used characterize these sections and were compared between samples. In neocortical (CX) coculture models, for amplitude, within the same chamber, the mean cross-correlation value is 0.28 ± 0.13 in the A-to-A section and 0.33 ± 0.15 in the B-to-B

section. Across chambers, the mean value is 0.17 ± 0.07 (A-to-B and B-to-A are the same due to the symmetry of the cross-correlation formula). For time lag, within the same chamber, the mean value is 0.43 ± 0.30 ms in the A-to-A section and 0.43 ± 0.20 ms in the B-to-B section. Across chambers, the mean value is -6.92 ± 33.00 ms in the A-to-B section. In hippocampal (HPC) coculture models, for amplitude, within the same chamber, the mean value is 0.25 ± 0.05 in the A-to-A section and 0.25 ± 0.06 in the B-to-B section. Across chambers, the mean value is 0.17 ± 0.06 (A-to-B and B-to-A are the same due to symmetry). For time lag, within the same chamber, the mean value is 0.35 ± 0.46 ms in the A-to-A section and 0.24 ± 0.35 ms in the B-to-B section. Across chambers, the mean value is -13.04 ± 20.95 ms in the A-to-B section. No significant difference is observed in amplitude or time lag between sections in both CX and HPC coculture models (Kruskal–Wallis test, $P > 0.05$).

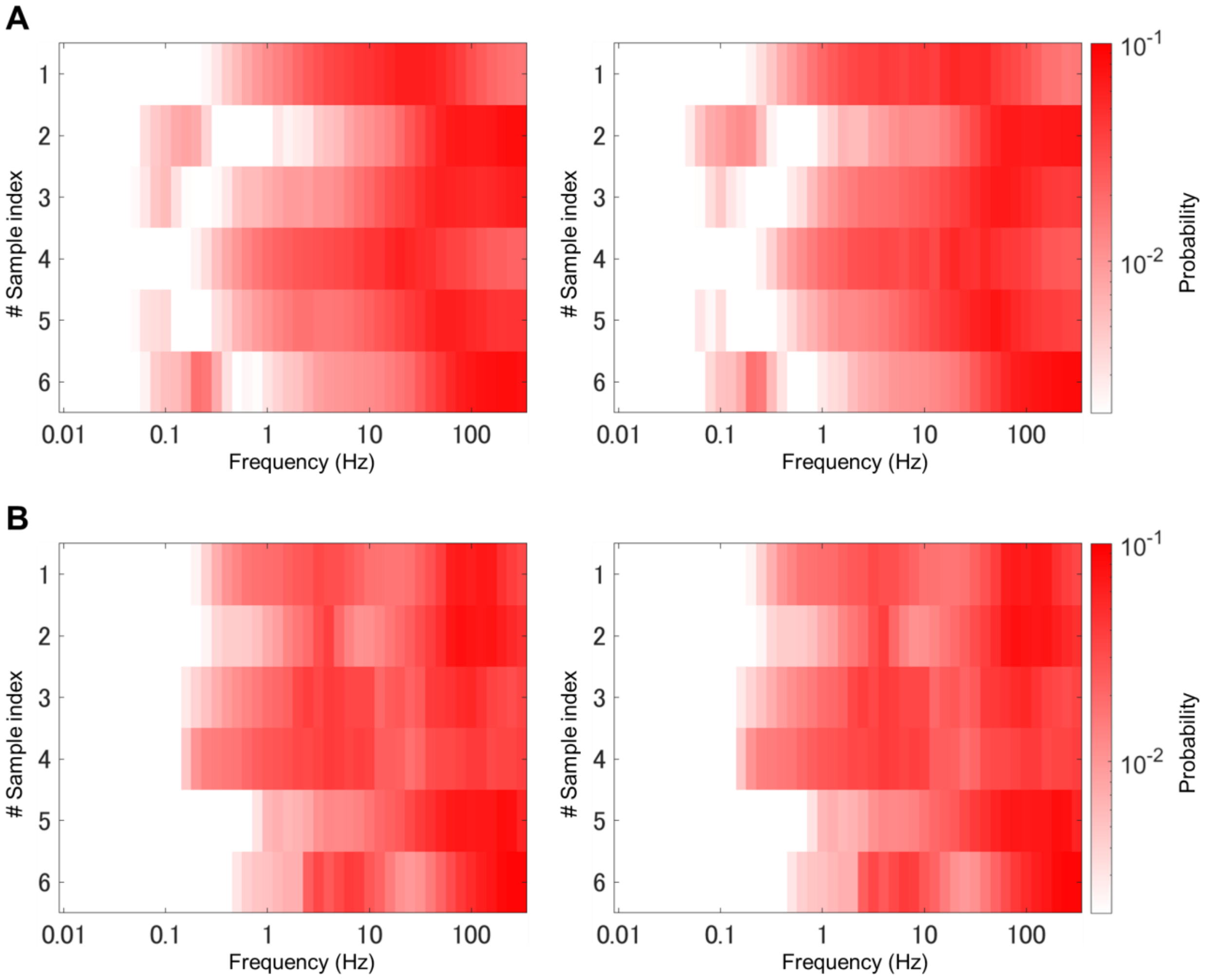

Supplementary Figure 6. Heatmaps of inverted inter-spike interval (ISI) in homogeneous cocultures. (**A**) is the heatmap of the neocortical (CX) cocultures, and (**B**) is the heatmap of the hippocampal (HPC) cocultures. ISI distributions from the two sides of the culture apparatus (chambers) are plotted in the right and left panels of (**A**) and (**B**). CX cocultures demonstrated a major peak around 20–40 Hz, which is considered low-gamma–like activity, and a minor peak between 0.1 Hz to 0.5 Hz, which is considered a slow-wave–like rhythm. HPC cocultures demonstrated two peaks—one at approximately 4–10 Hz (theta-wave–like activity) and the other at 100 Hz (high-gamma–like activity).

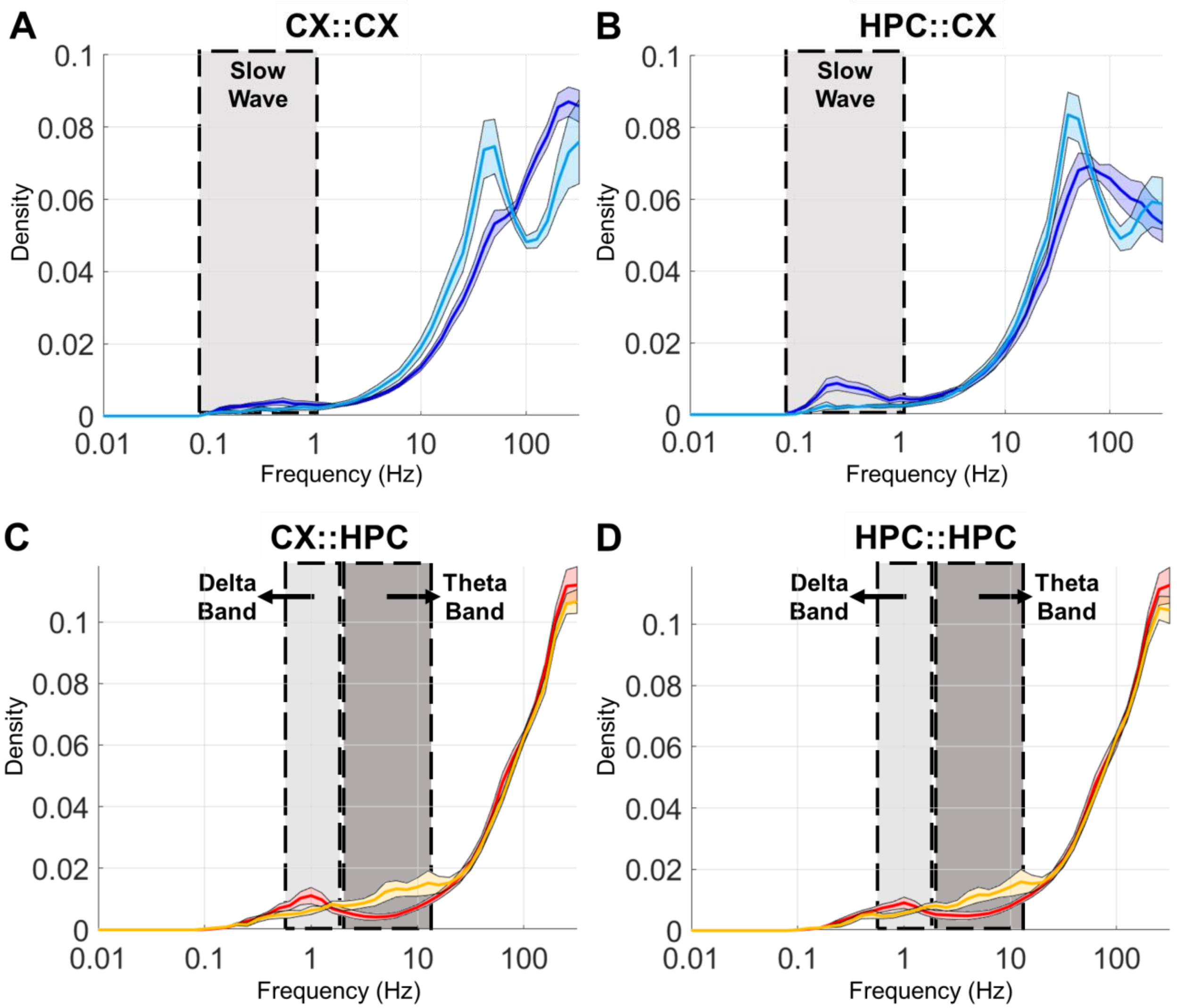

Supplementary Figure 7. Average inverted inter-spike interval (ISI) distribution of evoked responses in heterogeneous cocultures. (**A**) and (**B**) are the average ISI distributions of neocortical (CX) spike activity. (**C**) and (**D**) are the average ISI distributions of hippocampal (HPC) spike activity. The dark blue and red lines represent a first mode of activity coupling (slow wave and gamma in CX interacting with delta and high gamma in HPC). The light blue and yellow lines represent a second mode of activity coupling (low gamma in CX interacting with theta and high gamma in HPC). The shadowed area represents the standard error of each distribution. The response rate of Y evoked by stimulation delivered to X is expressed as X::Y, e.g., the direct response of the neocortical (CX) network to stimulation of the CX network is expressed as CX::CX, whereas the response of the hippocampal (HPC) network to stimulation of the CX network is expressed as CX::HPC.
